# Distortion of Visuo-Motor Temporal Integration in Apraxia: Evidence From Delayed Visual Feedback Detection Tasks and Voxel-Based Lesion-Symptom Mapping

**DOI:** 10.3389/fneur.2018.00709

**Published:** 2018-08-27

**Authors:** Satoshi Nobusako, Rintaro Ishibashi, Yusaku Takamura, Emika Oda, Yukie Tanigashira, Masashi Kouno, Takanori Tominaga, Yurie Ishibashi, Hiroyuki Okuno, Kaori Nobusako, Takuro Zama, Michihiro Osumi, Sotaro Shimada, Shu Morioka

**Affiliations:** ^1^Neurorehabilitation Research Center, Kio University, Nara, Japan; ^2^Graduate School of Health Science, Kio University, Nara, Japan; ^3^Department of Rehabilitation, Murata Hospital, Osaka, Japan; ^4^Cognitive-Neurorehabilitation Center, Setsunan General Hospital, Osaka, Japan; ^5^Rhythm-Based Brain Information Processing Unit, RIKEN CBS-TOYOTA Collaboration Center, RIKEN Center for Brain Science, Saitama, Japan; ^6^Department of Electronics and Bioinformatics, School of Science and Technology, Meiji University, Kanagawa, Japan

**Keywords:** apraxia, delayed visual feedback detection, forward model, multisensory temporal integration, subtraction lesion analysis, visuo-motor temporal integration, voxel-based lesion-symptom mapping

## Abstract

Limb apraxia is a higher brain dysfunction that typically occurs after left hemispheric stroke and its cause cannot be explained by sensory disturbance or motor paralysis. The comparison of motor signals and visual feedback to generate errors, i.e., visuo-motor integration, is important in motor control and motor learning, which may be impaired in apraxia. However, in apraxia after stroke, it is unknown whether there is a specific deficit in visuo-motor temporal integration compared to visuo-tactile and visuo-proprioceptive temporal integration. We examined the precision of visuo-motor temporal integration and sensory-sensory (visuo-tactile and visuo-proprioception) temporal integration in apraxia after stroke by using a delayed visual feedback detection task with three different conditions (tactile, passive movement, and active movement). The delay detection threshold and the probability curve for delay detection obtained in this task were quantitative indicators of the respective temporal integration functions. In addition, we performed subtraction and voxel-based lesion-symptom mapping to identify the brain lesions responsible for apraxia and deficits in visuo-motor temporal integration. The behavioral experiments showed that the delay detection threshold was extended and that the probability curve for delay detection was less steep in apraxic patients compared to controls (pseudo-apraxic patients and unaffected patients), only for the active movement condition, and not for the tactile and passive movement conditions. Furthermore, the severity of apraxia was significantly correlated with the delay detection threshold and the steepness of the probability curve in the active movement condition. These results indicated that multisensory (i.e., visual, tactile, and proprioception) feedback was normally temporally integrated, but motor prediction and visual feedback were not correctly temporally integrated in apraxic patients. That is, apraxic patients had difficulties with visuo-motor temporal integration. Lesion analyses revealed that both apraxia and the distortion of visuo-motor temporal integration were associated with lesions in the fronto-parietal motor network, including the left inferior parietal lobule and left inferior frontal gyrus. We suppose that damage to the left inferior fronto-parietal network could cause deficits in motor prediction for visuo-motor temporal integration, but not for sensory-sensory (visuo-tactile and visuo-proprioception) temporal integration, leading to the distortion of visuo-motor temporal integration in patients with apraxia.

## Introduction

Visuo-motor integration is an important function for motor control and motor learning (1, 2). It is largely supported by a neural mechanism known as the comparator or forward model (3, 4). The forward model provides stability to the motor system by predicting the sensory outcome of movements before actual sensorimotor feedback becomes available, providing a means of rapid online correction (5–9). When a mismatch occurs between motor prediction and actual sensory feedback, error signals are generated in order to correct/modulate the initial movement plan (5, 6, 10–16). Therefore, comparing motor signals and visual feedback to generate errors, i.e., visuo-motor integration, is the main function of the forward model. Furthermore, iterations of the comparison and matching processes of motor signals (including motor prediction) and sensory feedback generate a motor representation (e.g., kinesthetic memories, gesture engrams, visuo-kinesthetic engrams, and tool/object manipulation/use) (17–27). In addition, the neural basis for this process is the left frontal-parietal network, or more precisely, the left ventro-dorsal stream (19–31).

The main areas of the left ventro-dorsal stream are the inferior parietal lobule (IPL) and inferior frontal gyrus (IFG, including the ventral premotor cortex). Typically, limb apraxia is the result of higher brain dysfunction caused by stroke in the left IPL and left IFG. Apraxia was defined by Cubelli (32) as “the inability to perform specific and predefined actions or to carry out learned and purposeful movements, independently of sensory, motor and cognitive deficits that could impair the comprehension of the task, the recognition of the stimulus and the implementation of the response. Apraxia appears in daily activities and in standardized tests requiring actions to-be-performed on command and/or on imitation.” This current study followed the definition of apraxia by Cubelli (32). In the cognitive models of apraxia, the underlying cause has been ascribed to models rather than to a single deficit (33–35), i.e., there is not a single cause of apraxia. However, stroke patients with apraxia due to damage to the left IPL and left IFG areas have difficulties in performing motor learning tasks, imitating movements, and executing intransitive and transitive gestures, regardless of whether they are meaningless or meaningful (36–50). All of the tasks used in previous studies to examine patients with apraxia after stroke have included elements of visuo-motor integration; therefore, apraxia is thought to arise, in part, from an impairment of visuo-motor integration (17, 18, 44, 45, 47, 51, 52).

Unfortunately, in apraxia, no experimental study has examined whether the time window for visuo-motor integration in the brain is distorted. In apraxia, it is suggested that the sense of agency caused by the temporal integration of visual feedback and motor signals is lost (53), but no study has examined this issue. The time window for visuo-motor integration, i.e., the visuo-motor temporal integration function, which detects a temporal error between motor signals and visual feedback, can be evaluated quantitatively and objectively using a delayed visual feedback detection task (54–59). As apraxia involves an impairment of visuo-motor integration, the time window may also be distorted. Specifically, it may be difficult for subjects with apraxia to detect a temporal error between motor signals and visual feedback. It is important to emphasize that this hypothesis does not imply that the deficits in apraxia cannot occur due to a loss of stored motor representation, i.e., the stored knowledge of learned actions, in the technical reasoning function or in the other main functions. Rather, we believe that by quantitatively evaluating the time window for visuo-motor integration, which operates downstream to mechanisms involved in stored motor representation and technical reasoning, we can understand apraxia better.

The cognitive models for apraxia divide the symptoms generically referred to as apraxia into five categories (35). For example, the model predicts that a deficit of the action input lexicon causes “pantomime agnosia,” an impairment within the action semantic system causes “conceptual apraxia” (ideational apraxia without ideomotor apraxia), and a deficit of the visuomotor conversion mechanism causes “conduction apraxia” (ideomotor apraxia without ideational apraxia) (35). It is very important to recognize that apraxia is not a single disorder with a unique neuropsychological basis, but that various forms of apraxia exist (32). However, the current study focused on the presence of deficits in visuo-motor temporal integration underlying the symptoms generically referred to as apraxia. In other words, we were interested in whether various apraxia symptoms, other than an impairment of the imitation of meaningless gestures arising from a deficit of the visuo-motor conversion mechanism, were the result of deficits in visuo-motor temporal integration.

Therefore, the present study investigated visuo-tactile, visuo-proprioceptive, and visuo-motor temporal integration in left hemispheric stroke patients with or without apraxia under three conditions: tactile, passive, and active movement. Here, visuo-tactile temporal integration was defined as a function for detecting a temporal error between a tactile stimulus and visual feedback. Furthermore, visuo-proprioceptive temporal integration was defined as a function for detecting a temporal error between a proprioceptive stimulus and visual feedback. In the task, a visual feedback delay of 33–600 ms was incorporated into the image of the patient's non-paralyzed left hand, and the patient was asked whether or not there was a delay. The delay detection threshold (DDT, time delay in ms), which is the length of delay when the delay detection probability is 50%, and the steepness of the probability curve for delay detection, which will be referred to as “steepness” from this point onward, can be determined from this task. The DDT and steepness allowed us to examine the time window for multisensory (including motor signals) integration (58). The DDT indicated the extent to which the brain allowed a temporal discrepancy between different sensory modalities, including motor signals (motor prediction). Steepness indicated the mechanism by which the brain integrated multisensory signals. Thus, steepness would be increased if the judgment was more strict and precise. Therefore, shortening the DDT and/or increasing steepness represented highly sensitive multisensory (including motor signals) temporal integration, while prolonging the DDT and/or decreasing steepness represented poorly sensitive multisensory (including motor signals) temporal integration (55, 56). Subjects with apraxia have difficulties with visuo-motor integration (17, 18, 44, 45, 47, 51, 52); therefore, the current study hypothesized that patients with apraxia have normal visuo-tactile and visuo-proprioceptive temporal integration, but have deficits in visuo-motor temporal integration. Specifically, we hypothesized that the DDT would be prolonged and/or steepness would be decreased in patients with apraxia as compared to patients without apraxia.

Furthermore, in order to investigate the relationship between the severity of apraxia and multisensory (including motor signals) temporal integration and lesions, lesion analyses were carried out by subtraction and voxel-based lesion-symptom mapping (VLSM). In addition, imitation is performed by using visuo-motor conversion, while gesture by verbal instruction is processed through the action semantic system and by using procedural knowledge; therefore, lesions related to deficits in imitation and gesture may be subtly different. Thus, we also implemented VLSM based on deficits in imitation and pantomime.

## Materials and methods

### Participants

The participants were recruited from among patients receiving treatment and rehabilitation at Murata Hospital (Osaka, Japan) and Setsunan General Hospital (Osaka, Japan). The inclusion criterion was the occurrence of left hemispheric stroke. The exclusion criteria were a history of a mental disorder or developmental disability, a cognitive disorder (a cut-off score of 24 or lower on the Mini Mental State Examination [MMSE]), impaired language comprehension precluding the understanding of how to perform the experimental task, or impaired field of vision. In many previous studies, an MMSE cut-off score of 24 was used to exclude the effects of cognitive impairment (60–63). In addition, several studies have also revealed that there is a significant correlation between the MMSE score and the severity of apraxia in patients with cognitive impairment (64, 65). Therefore, in view of the important relationship between cognitive impairment and the symptoms of apraxia, we excluded patients with an MMSE score of 24 or less in the current study.

The presence of mental disorders was checked from the morbidity history in a database in which patient information was recorded, and patients with a history of a mental disorder (depression, schizophrenia, etc.) were excluded. This was because several previous studies revealed that patients with schizophrenia have difficulty in agency-attribution based on visuo-motor temporal integration, similar to the experimental task of the current study (66, 67).

Subjects with developmental disabilities, such as autism spectrum disorder and developmental coordination disorder, have been shown to have difficulties in sensory-sensory integration and sensory-motor integration (55, 68). Therefore, we also excluded patients with a history of developmental disabilities.

In consideration of the frequent occurrence of apraxia and aphasia (69), we evaluated the presence of aphasia in the patients using the standard language test of aphasia (SLTA) (70) and the supplementary tests for the SLTA (SLTA-ST) (71) to be certain that the patients could understand and respond to the experimental task. The SLTA is the only aphasia test standardized in Japan. The SLTA-ST is a battery of tests aimed at evaluating mild symptoms of aphasia and facilitates deeper testing than can be covered with the SLTA alone. The listening comprehension item of the SLTA is a 6-grade evaluation, and grades 5 and 6 indicate that there is no impairment of listening comprehension. The SLTA-ST includes Yes-No response items to which an answer of “Yes” or “No” is required. Therefore, as criteria to ensure that the patients could understand and respond to the experimental task, the current study included patients whose listening comprehension items of the SLTA were grade 5 or 6 and with 100% correct answers to the Yes-No response items of the SLTA-ST. We excluded patients who did not meet these criteria.

As a result, 22 patients with left hemispheric stroke (average age ± standard deviation [SD] of 67.5 ± 15.5 years, male = 15, all right-handed) consented to participate in the present study. The study received approval from the ethics committee of the Faculty of Health Sciences at Kio University (approval number: H27-16). The demographic characteristics of the patients are shown in Table 1.

**Table 1:** Patient demographic characteristics and apraxia assessment results.

**Patient no**.	**Education (years)**	**MMSE**	**Disease duration (days)**	**Handedness**	**Left upper limb function**		**Body awareness of left upper limb**		**Apraxia**			**Group**
					**Motor function**	**Sensory function**	**Asomato gnosia**	**Sense of ownership**	**Imitation**	**Gesture**	**Total score**	
1	18	25	61	right	10	6	none	3	5	2	7	apraxia
2	21	25	280	right	10	6	none	3	4	1	5	apraxia
3	18	25	68	right	10	6	none	3	0	1	1	apraxia
4	21	30	42	right	10	6	none	3	2	3	5	apraxia
5	18	27	397	right	10	6	none	3	4	3	7	apraxia
6	18	26	18	right	10	6	none	3	4	2	6	apraxia
7	12	25	1,126	right	10	6	none	3	4	3	7	apraxia
Mean	18.0	26.1	284.6		10.0	6.0		3.0	3.3	2.1	5.4	
SD	2.8	1.7	367.9		0.0	0.0		0.0	1.6	0.8	2.0	
8	18	27	338	right	10	6	none	3	7	4	11	pseudo-apraxia
9	18	25	631	right	10	6	none	3	7	3	10	pseudo-apraxia
10	12	25	35	right	10	6	none	3	5	4	9	pseudo-apraxia
11	18	29	123	right	10	6	none	3	6	3	9	pseudo-apraxia
12	18	27	19	right	10	6	none	3	6	3	9	pseudo-apraxia
13	18	28	523	right	10	6	none	3	7	4	11	pseudo-apraxia
Mean	17.0	26.8	278.2		10.0	6.0		3.0	6.3	3.5	9.8	
SD	2.2	1.5	237.5		0.0	0.0		0.0	0.7	0.5	0.9	
14	18	26	54	right	10	6	none	3	7	5	12	unaffected
15	22	28	48	right	10	6	none	3	7	5	12	unaffected
16	18	29	628	right	10	6	none	3	7	5	12	unaffected
17	18	28	47	right	10	6	none	3	7	5	12	unaffected
18	21	25	120	right	10	6	none	3	7	5	12	unaffected
19	22	30	556	right	10	6	none	3	7	5	12	unaffected
20	18	25	47	right	10	6	none	3	7	5	12	unaffected
21	18	29	15	right	10	6	none	3	7	5	12	unaffected
22	18	25	48	right	10	6	none	3	7	5	12	unaffected
Mean	19.2	27.2	173.7		10.0	6.0		3.0	7.0	5.0	12.0	
SD	1.7	1.9	225.7		0.0	0.0		0.0	0.0	0.0	0.0	

*MMSE, Mini-Mental State Examination; SD, Standard deviation*.

*Handedness was determined according to the Edinburgh Handedness Inventory (72)*.

*Left upper limb function was measured with the Stroke Impairment Assessment Set (SIAS)*.

*Body awareness of the left upper limb was measured by the Verbal Asomatognosia and Somatoparaphrenia Assessment (VASA) and a 7-point Likert scale on body ownership*.

*Apraxia was evaluated using the apraxia screen of the TULIA (AST)*.

*Group (apraxia; pseudo-apraxia; unaffected) shows grouping based on the AST results*.

### Procedure

#### Neurological assessments

All patients underwent a neurological assessment of the left (unaffected side of the body) upper limb and performed the experimental task using the left limb over a 2-day period.

The Stroke Impairment Assessment Set (SIAS) (73) was used to exclude patients with a paralyzed left upper limb or sensory loss in that limb. Moreover, the motor function test of the SIAS included two different upper limb motor function tests: the knee-mouth test and finger-function test. All participants were evaluated on a 6-point scale (0–5 points) with 0 points being no muscle contraction and no movement observed, and 5 points being muscular strength and coordination comparable to the non-paralyzed side, with limb movements possible. Therefore, the total possible score of the knee-mouth and finger-function tests was 10 points, indicating that upper limb motor function was normal. The sensory evaluation test of the SIAS was performed with the upper limbs by applying a tactile stimulus (a brush) to the palm of the hand and a proprioceptive stimulus by passive movement of the thumb and index finger. Each test was evaluated using a score from 0 to 3 points, with a loss of sensation being 0 points and normal sensation being 3 points. In addition, patients with abnormal perception and pain were rated at 2 points. Therefore, if the total score of the tactile and proprioceptive tests was 6 points, it indicated that there was no disorder and/or abnormal feeling of tactile sensation and proprioceptive sensation in the hand. The results of the SIAS indicated that all patients had a motor function score of 10 points for their left upper limb and a sensory function score of 6 points (Table 1); thus, none of the upper left limbs of the patients were paralyzed or showed sensory loss.

In addition, the Verbal Asomatognosia and Somatoparaphrenia Assessment (VASA) (74) was used to exclude patients with asomatognosia or somatoparaphrenia involving the left upper limb. Further, body ownership of the hand was evaluated using the following 7-item scale: 3, I think it is my hand to the utmost extent; 2, I think it is my hand to a moderate extent; 1, I think it is my hand to a minimal extent; 0, I cannot say either way; −1, I think it is not my hand to a minimal extent; −2, I think it is not my hand to a moderate extent; and −3, I think it is not my hand to the maximum extent. The results of the VASA verified that none of the patients had asomatognosia or somatoparaphrenia involving their left upper limb. In addition, the 7-point Likert scale of body ownership indicated normal ownership of the left upper limb of the patients (Table 1).

Furthermore, apraxia in the left upper limb was evaluated using an apraxia screen created from the 12 following test items extracted from the Test of Upper Limb Apraxia (TULIA [AST]) (75, 76): one imitation of a meaningless intransitive gesture, one imitation of a meaningful intransitive gesture, five imitations of transitive (tool-related) gestures, two meaningful intransitive gestures from verbal instructions, and three pantomimes of transitive (tool-related) gestures from verbal instructions. The AST has high specificity (93%) and sensitivity (88%) and good test–retest reliability (76, 77). On the basis of the AST scoring criteria, 0 points were given as a “Fail” if the following were observed: appearance of a body part as an object error, considerable spatial errors, extra movements, omissions, false end position, substitutions, perseverations, or amorphous or seeking movements not related to the desired gesture. On the basis of the AST scoring criteria, 1 point was given as a “Pass” if the following were observed: normal movement, slight slowdown or discrete spatial errors (e.g., diminished amplitude), discrete extra movements or omissions, corrected brief substitutions or perseverations. The maximum score on the AST was 12 points. A score of 12 points indicated no apraxia, a score of <9 points indicated apraxia, and a score of <5 points indicated severe apraxia. The 22 left limbs of the patients were classified into three groups according to their AST-based apraxia score. Non-apraxic limbs with a perfect score of 12 points on the AST formed the unaffected group; limbs with a score of ≥9 points and ≤ 11 points that exhibited apraxia-like symptoms, but were free of apraxia, formed the pseudo-apraxic group; and limbs with a score of <9 points (the cut-off for apraxia on the AST) formed the apraxic group. On the basis of the standards described above, 9 patients were categorized into the unaffected group (male = 7, average age ± SD = 69.3 ± 13.9 years, range: 43–87 years), 6 into the pseudo-apraxic group (male = 5, average age ± SD = 57.2 ± 16.5 years, range: 30–77 years), and 7 into the apraxic group (male = 3, average age ± SD = 74.0 ± 11.7 years, range: 53–88 years) (Table 1). This classification took into consideration the possibility that there may be a difference in the index obtained in the experimental task between the group of patients (pseudo-apraxic group) who were not diagnosed at a level indicating apraxia, but had slightly apraxic symptoms, and a group of patients with no apraxic symptoms (unaffected group).

All evaluations were performed by neurologists, physical therapists, and occupational therapists with clinical experience of neurological assessments.

#### Experimental task

##### Set-up

**Figure 1:**
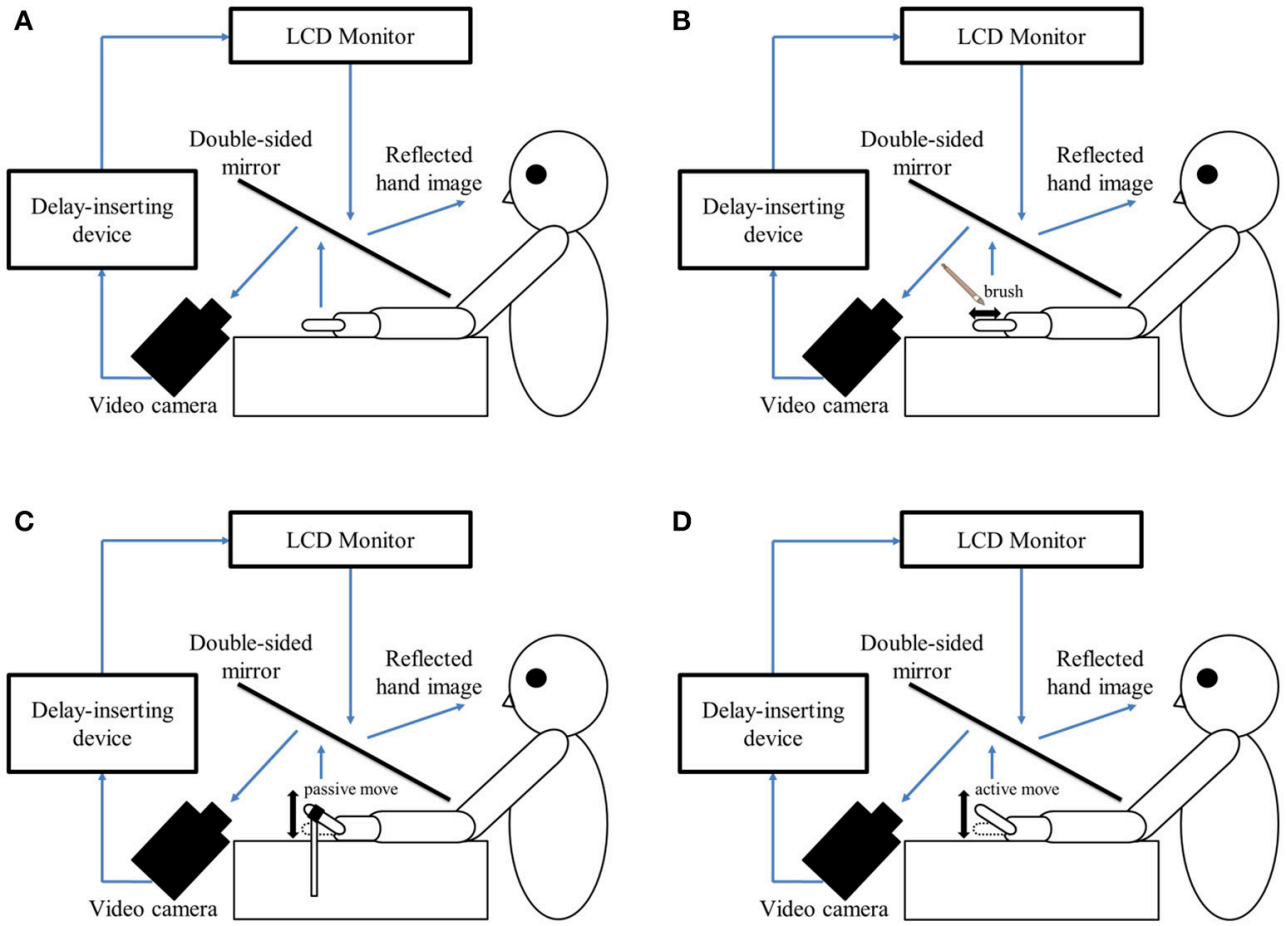
Experimental setup and experimental task. **(A)** Setup of the experimental task. The patient placed their left hand under a two-way mirror. The patient was able to see their left hand projected on a two-way mirror. **(B)** Tactile stimulation condition. The patient's left index finger was stimulated tactilely by a brush. **(C)** Passive movement condition. The patient's left index finger was moved passively. **(D)** Active movement condition. The patient moved their left index finger under their own volition. The finger was filmed by a video camera under all conditions **(B–D)**. Visual feedback delay was achieved using a hardware device. The patient observed the reflected image of their delayed finger displayed on an LCD monitor. For each trial of each stimulation condition, the patient was instructed to reply orally “delayed” or “not delayed” by the forced-choice method immediately following the trial.

In this study, a similar experimental design as that reported by Shimada et al. (58) was used in the testing laboratory of each hospital (Figure 1A). The patient's left hand was placed under a two-way mirror, and they were unable to see their hand directly. The reflection of the hand in a two-way mirror was imaged with a video camera (FDR-AXP35; Sony, Tokyo, Japan). The image of the videoed hand was reflected from an installed monitor (LMD-A240, Sony) onto the two-way mirror via a video delay device (EDS-3306; FOR-A YEM Eletex, Tokyo, Japan), and the patient observed the image of their own hand reflected in the mirror. The intrinsic delay of the visual feedback in this experimental setting was 33.71 ms as measured by a time-lag check device (EDD-5200; FOR-A YEM Eletex, Tokyo, Japan). Seven delay conditions of 33, 100, 200, 300, 400, 500, and 600 ms were used. Thus, the patient observed the delayed visual feedback of their own hand vs. the tactile stimulation, passive motion, and active motion of their own hand in real-time. In addition, the experimental design included a blackout curtain so that the patient could not see outside the experimental chamber.

##### Task

The delayed visual feedback detection tasks were performed on the left hand of all patients using the experimental setup for the three stimulation conditions (tactile, passive, and active movement) and the seven delay conditions (33–600 ms). The patients had to answer orally whether or not there was a visual feedback delay compared to their own hand sensation/movement, in a forced-choice manner, immediately after the trial.

In the tactile stimulation condition (Figure 1B), tactile stimulation was performed by the experimenter with a brush on the patient's index finger with one stroke from the base to the tip of the finger, and back again. In the passive movement condition (Figure 1C), passive extension (rising) and flexion (lowering) movements (movement of the index finger away from the supporting surface and returning back again to the supporting surface) were performed on the patient's index finger. The passive extension (rising)-flexion (lowering) movements were performed by the experimenter raising and lowering a rod fixed to a hook and loop fastener (e.g., Velcro®) on the patient's index finger. The tactile stimulations and passive movements were performed by the same experimenter for all patients in order to ensure that all trials were performed with uniform stimulation. The experimenter was familiar with the purpose and tasks of this experiment, but was not informed of the patients' symptoms to avoid a bias in that the amount of stimulation might be changed by that knowledge. In the active movement condition (Figure 1D), extension (raising)-flexion (lowering) movements of the index finger were performed based on each patient's own volition. The patient was able to start the movement according to their own volition after the experimenter had informed them orally of the start of a trial. In addition, when the patient performed the wrong movement (i.e., movement other than extension-flexion of the index finger), the trial was performed again.

The seven delay conditions of one trial were treated as one set, and seven sets were performed for each stimulation condition. The presentation order of the delay conditions in a single set was randomized. In addition, the order of the three stimulation conditions was also randomized across patients. Three stimulation conditions × 7 delay conditions × 7 sets were conducted for each patient's left hand, resulting in a total of 147 trials. A 10 s rest period was set between each trial. In addition, a 3-min break period was set between each stimulation condition.

#### Data analysis

The three groups were compared in terms of sex using the chi-square test for independence. Age was compared by one-way analysis of variance (ANOVA) because a normal distribution and homoscedasticity were confirmed with the Shapiro–Wilk test and Levene's test. Duration of education (years), general cognitive function (MMSE), and period of time after stroke (days) were tested by the Kruskal-Wallis test because a normal distribution was not detected with the Shapiro–Wilk test.

##### Experimental data analysis

All 22 patients completed the visual feedback delay detection tasks for all conditions. We calculated the delay detection probability for each condition and patient. In order to examine the differences in the shapes of the delay detection probability curves, a logistic curve was fitted to the patient's responses (58, 78) according to the following formula: P(t) = 1/1 + exp(-*a*[t–tDDT]), where t was the visual feedback delay length, P(t) was the probability of delay detection, *a* indicated the steepness of the fitted curve, and tDDT indicated the observer's DDT, representing the delay length at which synchrony and asynchrony judgment probabilities were equal (50%). In our experiment, t served as an independent variable, and P(t) was the observed value. Fitting was performed using a nonlinear least squares method (a trust-region algorithm), provided by the Curve Fitting toolbox in MATLAB R2014b (MathWorks, Inc., Natick, MA, USA), to estimate *a* (signifying the steepness of the logistic curve) and tDDT.

As the values for the perceived DDT had a normal distribution in Shapiro–Wilk tests, ANOVA for split-plot factorial designs was used for comparisons between three groups and the three stimulation conditions in the DDT. The values for perceived steepness did not have a normal distribution in Shapiro–Wilk tests; therefore, the Kruskal-Wallis test was used for intergroup comparisons of steepness. Mann–Whitney U tests were used for *post-hoc* analyses. In addition, the comparison between the stimulus conditions of steepness used the Friedman test and Wilcoxon signed-rank test for *post-hoc* analyses. Correlation analysis was performed using Spearman's correlation coefficient by a rank test between the severity of apraxia and DDT/steepness.

The significance level was set at *p* < 0.05 for all statistical analyses, and the Bonferroni correction was used to adjust for multiple comparisons. All statistical analyses were performed using SPSS, version 24 (SPSS, Chicago, IL, USA).

##### Lesion analysis

Lesion analyses were based on the cranial magnetic resonance imaging (MRI) scans of patients conducted in their clinical stage. MRI examinations of the patients were performed at the hospitals where they were examined. Of the 22 participants, 19 imaging findings were collected. No imaging findings were collected for two patients (numbers 8 and 11) in the pseudo-apraxic group and one patient (number 18) in the unaffected group.

Subsequently, using the collected MRI data (*n* = 19), lesion analyses and spatial normalizations were performed according to the following procedure. Two experienced investigators (RI and YT) manually delineated the boundaries of the ischemic lesion on anonymized imaging scans using MRIcron (79), and the AC-PC line was set automatically using a custom MATLAB script. The ischemic lesion was delineated on fluid-attenuated inversion recovery (FLAIR) MRI sequences (1.5 T MRI: repetition time = 4,500 ms, echo time = 108 ms, field of view = 480 × 480 mm^2^, slice thickness = 6 mm, voxel size = 0.6875 × 0.6875 × 8 mm^3^) that had been performed at 4–7 days post-stroke. To assure sufficient spatial normalization, we used T2-weighted images (1.5 T MRI: repetition time = 4,500 ms, echo time = 108 ms, field of view = 480 × 480 mm^2^, slice thickness = 6 mm, voxel size = 0.4583 × 0.4583 × 8 mm^3^) as the anatomical scans. To avoid observer bias, two raters were blinded to the clinical parameters and apraxia scores (AST) during image analysis. The MRI scans and ischemic lesion shape were transferred into a stereotaxic space using the normalization algorithm of SPM8 and the Clinical Toolbox for SPM8 (80). Using the MR normalized algorithm of Clinical Toolbox, the MRI-derived lesion shape and the MRI scans were transformed to the T1 template based on older individuals with a resampled voxel size of 1 × 1 × 1 mm^3^ (80).

The ischemic lesion was delineated manually on a pathologic scan, i.e., the FLAIR sequences, as described above. To remove the jagged edges that were created during lesion delineation, the lesion was smoothed with 8 mm full width at half maximum and 0.5 threshold. The pathological scan was used to coregister the lesion map to the T2-weighted anatomical scan. Then, a unified-segmentation normalization algorithm was applied to the anatomical scan by assuming *a priori* maps of gray matter, white matter, and cerebral spinal fluid in the brain (80–82). The modality was FLAIR, the template mask was not set, the bounding box was set to 2 × 3 double, intermediate images were set to define the origin automatically as False, and normalization was conducted. The normalized T1 scan and 3 mm smoothed lesion were resliced and the normalized lesion was binarized (80). The normalized lesion map was then analyzed with non-parametric mapping software implemented in the MRIcron software package (80). Individual normalized lesion maps were subjected to intensity filters (minimum threshold, 100; maximum threshold, 255). Individual lesion volumes were recorded after filtering.

We performed subtraction analysis to determine whether the lesion overlap of the patients in the apraxic group differed from the lesion overlap of patients without apraxia (pseudo-apraxic and unaffected groups). We created an overlap image of the apraxic group (*n* = 7) and of the pseudo-apraxic and unaffected groups (*n* = 12). The subtraction analysis subtracted the lesion overlap of the pseudo-apraxic and unaffected groups from the lesion overlap of the apraxic group (83).

As a second approach for lesion analysis, non-parametric mapping, a statistical package implemented in MRIcron, was used (79). To correlate the individual AST scores (severity of apraxia) and experimental variables (DDT and steepness) with the individual lesion of each patient with left hemispheric stroke, we administered VLSM to identify those lesioned voxels that were significantly associated with deficits in the respective task. In addition, since the AST was composed of imitation and gesture tests, VLSM was also implemented for imitation and gesture deficits, respectively. Regarding the comparison of the lesion patterns of left hemispheric stroke patients with and without apraxia, only voxels that were damaged in at least 20% of patients were included in VLSM. The Brunner-Munzel test was used to compare each voxel, and considered significant when passing a statistical threshold of *p* < 0.05, corrected by the false discovery rate (47, 84, 85). Anatomical localization was assessed using automatic anatomical labeling (86) implemented in MRIcron.

In addition, correlation analysis between lesion volume, severity of apraxia (AST score), and experimental variables (DDT and steepness) was conducted using Spearman's correlation coefficient by a rank test, taking account of the relationship between lesion volume and each measured value.

## Results

The three groups showed no significant differences in sex [$\chi_{\left(0.95\right)}^{2}$ = 5.991, χ^2^ = 3.086, *p* = 0.214], age [*F*_(2, 21)_ = 2.092, *p* = 0.151], educational background (χ^2^ = 3.109, *p* = 0.211), general cognitive function (χ^2^ = 1.417, *p* = 0.492), or length of time since stroke (χ^2^ = 0.526, *p* = 0.769).

### Experimental data analysis

**Figure 2:**
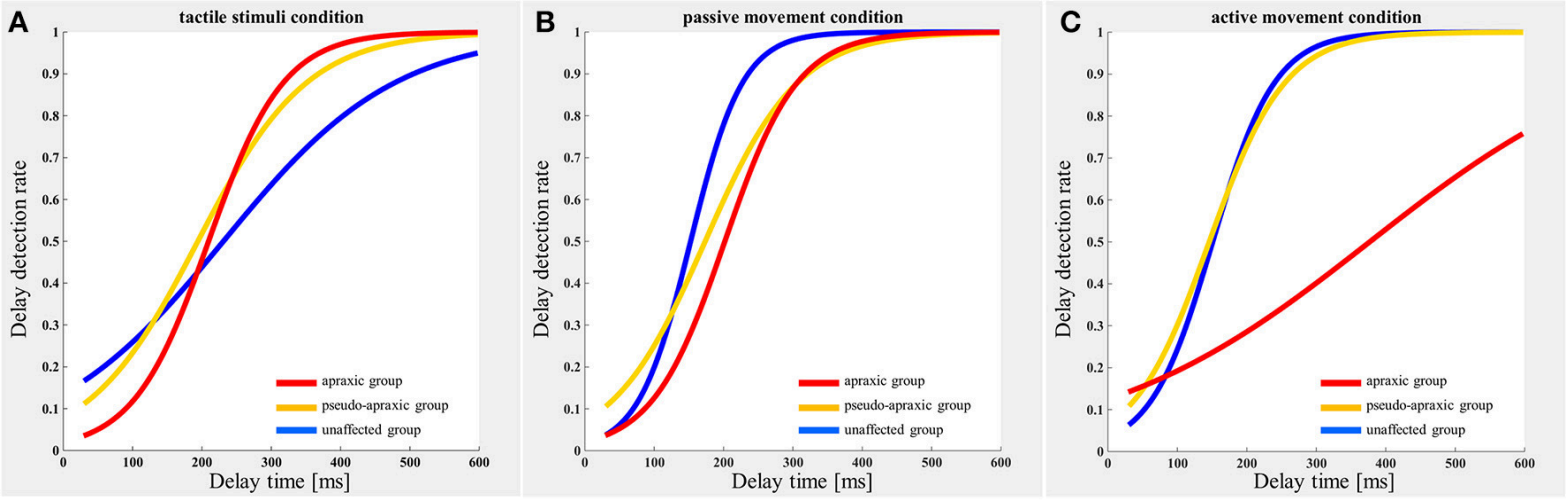
Delay detection probability curve for each condition in each group. Blue, unaffected group (*n* = 9); yellow, pseudo-group (*n* = 6); and red, apraxic group (*n* = 7). **(A)** Tactile stimuli condition. The delay-detection probability curve shows a similar shape in each group. **(B)** Passive movement condition. The logistic curve forms were similar to those in **(A)**. **(C)** Active movement condition. The judgment curve for active movements of the apraxic group showed a different shape from the other groups.

The delay detection probability curve for each group is shown in Figure 2. The three groups had a similar probability curve for the tactile stimuli (Figure 2A) and passive movement conditions (Figure 2B). The apraxic group produced a probability curve in response to active movements that differed markedly from the probability curves for the unaffected and pseudo-apraxic groups (Figure 2C).

**Figure 3:**
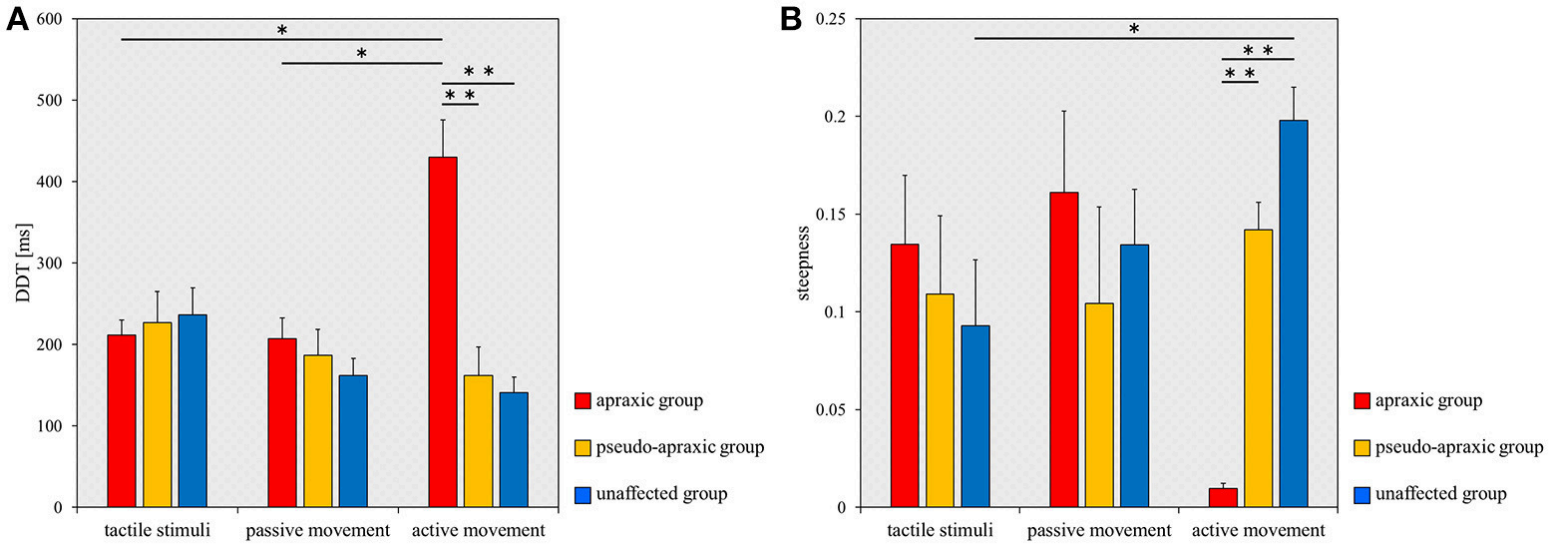
Mean DDT or steepness under each stimulus condition in each group. DDT, the delay detection threshold for the detection of delayed visual feedback. Steepness, the steepness of the probability curve for the detection of delayed visual feedback. The horizontal axis shows each stimulus condition. Blue bar, unaffected group (*n* = 9); yellow bar, pseudo-apraxic group (*n* = 6); and red bar, apraxic group (*n* = 7). Error bars represent standard error of the mean. **(A)** Comparison of the mean DDT of each group in each stimulus condition. **(B)** Comparison of the mean steepness of each group in each stimulus condition. *p < 0.05; **p < 0.01, Bonferroni-corrected.

For the DDT, there was no main effect [*F*_(2, 38)_ = 2.602; *p* = 0.087] of stimulation condition according to ANOVA for split-plot factorial design, but there was a significant interaction effect [*F*_(4, 38)_ = 8.285; *p* < 0.001] of group and stimulus condition. The comparisons between groups and stimulation conditions for the DDT are shown in Figure 3A. A multiple comparisons test using Tukey's method showed that the DDT for active movements was significantly longer in the apraxic group than in the pseudo-apraxic and unaffected groups (vs. pseudo-apraxic, *p* < 0.001; vs. unaffected, *p* < 0.001). There was no significant difference in the active movement condition between the pseudo-apraxic and unaffected groups (*p* = 0.914). There was no significant difference in the DDT for the tactile and passive movement conditions among the three groups (Figure 3A).

In addition, subsequent analyses using paired *t*-tests and Bonferroni's correction showed that the DDT in the apraxic group was significantly longer in the active movement condition than in the tactile and passive movement conditions (vs. tactile stimuli, *t* = −4.461; *p* = 0.013, Bonferroni-corrected; vs. passive movements, *t* = −3.968; *p* = 0.022, Bonferroni-corrected). There was no significant difference in the DDT of the apraxic group between the tactile stimuli and passive movement conditions (*t* = 0.222; *p* = 2.495, Bonferroni-corrected). There was no significant difference in the DDT among the stimulus conditions in the pseudo-apraxic and unaffected groups (Figure 3A).

Similarly, we found significant differences in the steepness of the delay detection probability curve among the three groups in the active movement condition (*p* < 0.001; Figure 3B). Subsequent analyses showed that steepness decreased significantly in the apraxic group compared to the pseudo-apraxic group (*p* = 0.003, Bonferroni-corrected) and unaffected group (*p* < 0.001, Bonferroni-corrected). There was no significant difference in the steepness of the active movement condition between the pseudo-apraxic and unaffected groups (*p* = 0.108, Bonferroni-corrected). There was no significant difference in steepness among the three groups in the tactile and passive movement conditions (tactile stimuli, *p* = 0.526; passive movements, *p* = 0.729).

A significant difference in steepness among the three stimulation conditions was found in the unaffected group (*p* = 0.035; Figure 3B). Subsequent analyses showed that steepness in the unaffected group was significantly lower in the tactile condition than in the active movement condition (*p* = 0.040, Bonferroni-corrected). There was no significant difference in the unaffected group between the tactile and passive movement conditions (*p* = 0.231, Bonferroni-corrected) as well as between the passive and active movement conditions (*p* = 0.1000, Bonferroni-corrected). There was no significant difference in steepness among the stimulation conditions in the pseudo-apraxic and apraxic groups (pseudo-apraxic group, *p* = 0.846; apraxic group, *p* = 0.066).

**Figure 4:**
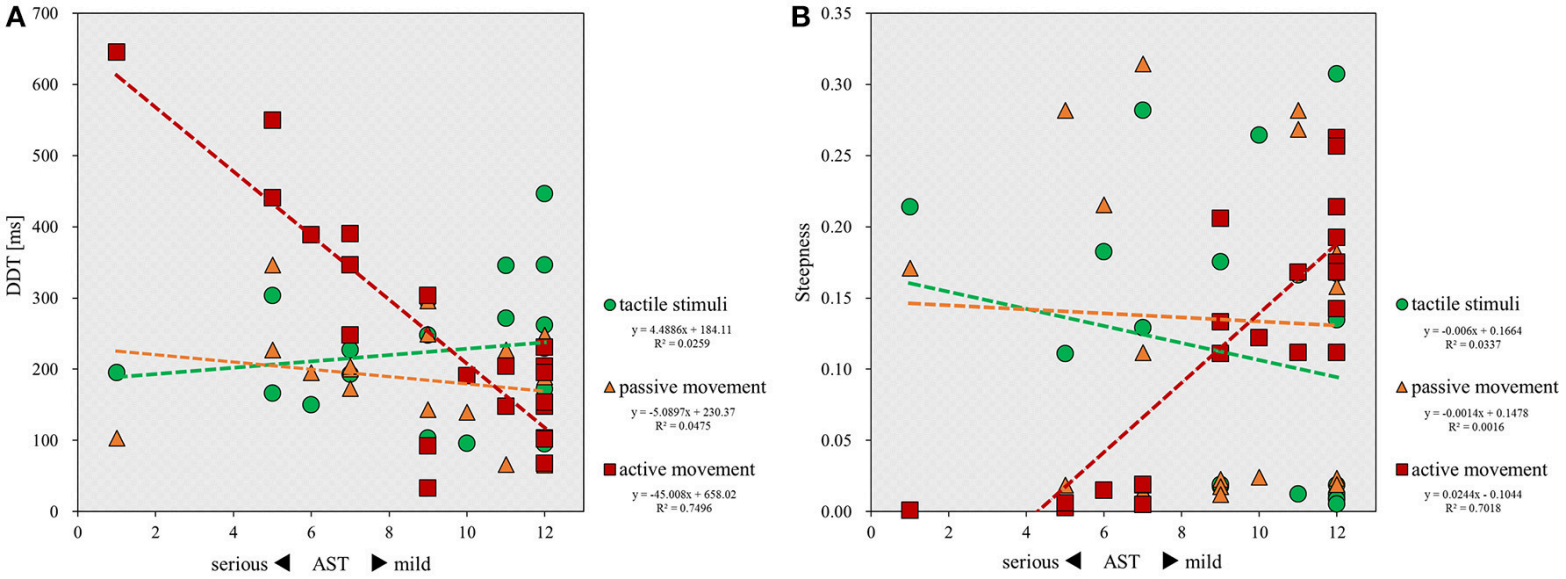
Scatterplot showing the relationship between the severity of apraxia and DDT or steepness for all patients in each stimulus condition. DDT, the delay detection threshold for the detection of delayed visual feedback. Steepness, the steepness of the probability curve for the detection of delayed visual feedback. The horizontal axis shows the AST score for the severity of apraxia. **(A)** Correlation between the severity of apraxia and the DDT of each stimulus condition (*n* = 22). **(B)** Correlation between the severity of apraxia and the steepness of each stimulus condition (*n* = 22).

Finally, correlation analyses showed that there was a significant inverse correlation between the severity of apraxia and DDT in the active movement condition (*r* = −0.705; *p* < 0.001), but not in the tactile (*r* = 0.112; *p* = 0.620) and passive movement conditions (*r* = −0.262; *p* = 0.239; Figure 4A). Similarly, a significant correlation was observed between the severity of apraxia and steepness in the active movement condition (*r* = 0.850; *p* < 0.001), but not in the tactile (*r* = −0.227; *p* = 0.310) and passive movement conditions (*r* = 0.002; *p* = 0.994; Figure 4B).

### Lesion analysis

**Figure 5:**
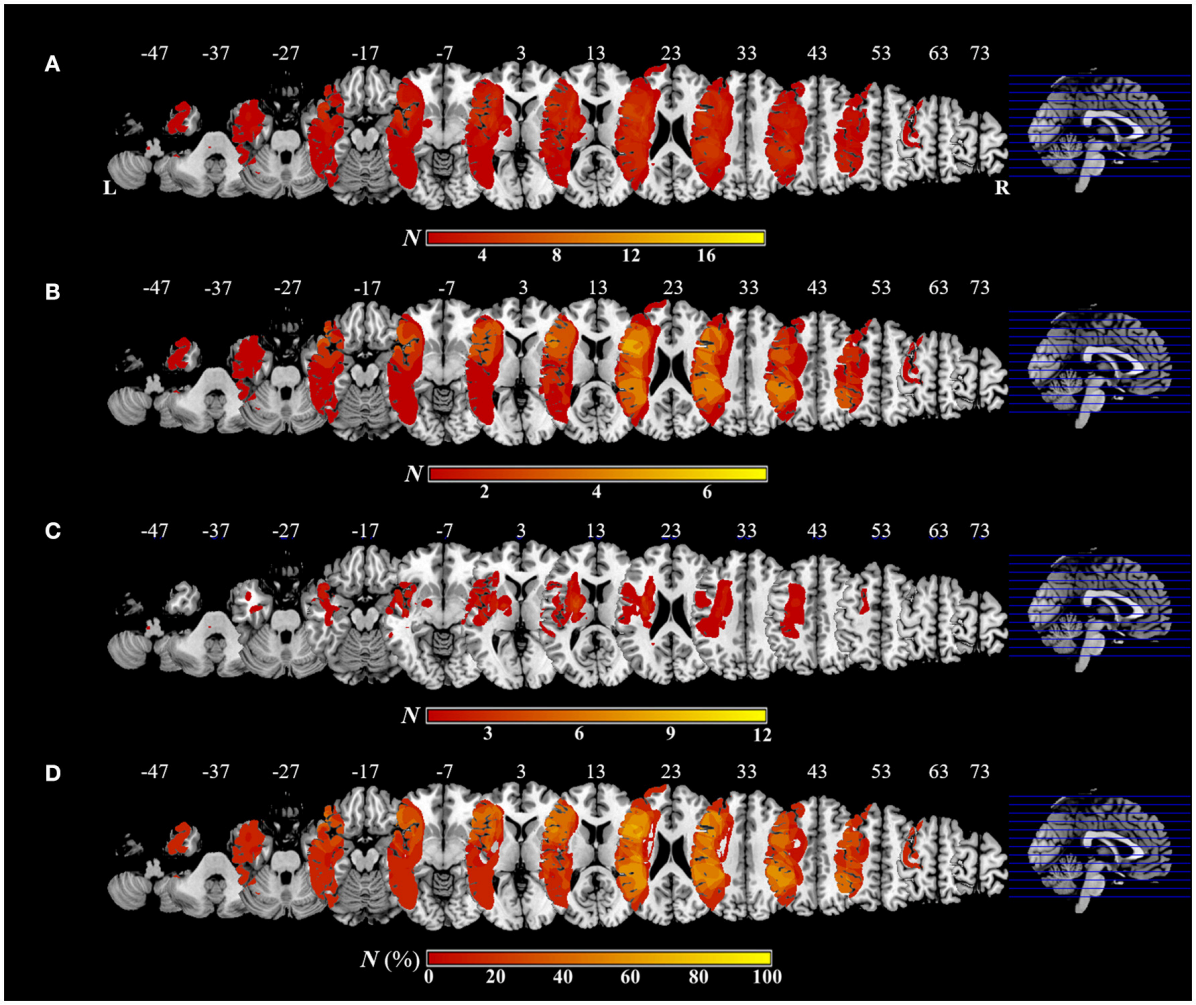
Overlap and distribution of ischemic lesions of all patients and each group, and subtraction lesion mapping. The number of overlapping lesions is illustrated by different colors indicating increasing frequency from red to yellow. Montreal Neurological Institute z coordinates of each transverse section are given. L, left hemisphere; R, right hemisphere; N, number of individuals with a lesion in a given voxel; N (%), number of lesion overlaps after subtraction, expressed as a percentage. **(A)** Overlap and distribution of ischemic lesions of all patients (*n* = 19). **(B)** Overlap and distribution of ischemic lesions of the apraxic group (*n* = 7). **(C)** Overlap and distribution of ischemic lesions of the pseudo-apraxic and unaffected groups (*n* = 12). **(D)** After subtraction of lesion overlap of the patients in the pseudo-apraxic and unaffected groups from the lesion overlap of the patients in the apraxic group, lesioned voxels in the left precentral gyrus, left inferior frontal gyrus (including the opercular triangular regions), left rolandic operculum, left insular, and left inferior parietal lobule (including the left supramarginal gyrus) were associated with apraxia after stroke.

Figure 5 shows the distribution and overlap of lesions in all patients and each group, and the results of the subtraction analyses. The peak coordinates and the number of lesion overlaps of all patients, each group, and after subtraction are shown in Table 2. Larger clusters (71%) of lesioned voxels in the left precentral gyrus, left IFG (including the opercular and triangular regions), left rolandic operculum, left insular, and left IPL (including the supramarginal gyrus) were associated with apraxia after stroke. Subsequently, the next largest clusters (57%) of lesioned voxels were in the left superior and middle frontal gyrus, left postcentral gyrus, left superior parietal lobule, and left angular gyrus, as well as in the left superior and middle temporal gyrus, and were associated with apraxia after stroke.

**Table 2:** Voxel-wise lesion overlapping and subtraction analyses.

**Group**	**Area**	**Max (N, %)**	**MNI coordinates (x, y, z)**
All patients (*n* = 19)	Frontal_Inf_Oper_L	7	−55, 15, 18
	Frontal_Inf_Tri_L	7	−53, 20, 17
	Rolandic_Oper_L	7	−41, −17, 24
	Precentral_L	6	−42, 5, 23
	Insula_L	6	−31, 13, 10
	Postcentral_L	6	−49, −19, 28
	Parietal_Inf_L	6	−44, −26, 36
	SupraMarginal_L	6	−56, −27, 26
Apraxic group (*n* = 7)	Precentral_L	5	−42, 5, 23
	Frontal_Inf_Oper_L	5	−54, 14, 17
	Frontal_Inf_Tri_L	5	−48, 19, 17
	Rolandic_Oper_L	5	−38, −20, 20
	Insula_L	5	−37, −20, 20
	Parietal_Inf_L	5	−44, −26, 36
	SupraMarginal_L	5	−46, −25, 33
	Frontal_Sup_L	4	−29, 25, 33
	Frontal_Mid_L	4	−33, 33, 17
	Postcentral_L	4	−50, −8, 19
	Parietal_Sup_L	4	−31, −49, 51
	Angular_L	4	−40, −55, 22
	Temporal_Sup_L	4	−42, −40, 19
	Temporal_Mid_L	4	−42, −54, 22
Pseudo-apraxic and unaffected groups (*n* = 12)	Putamen_L	5	−24, −3, 11
	Insula_L	4	−33, −15, 17
	Rolandic_Oper_L	3	−36, −7, 15
	Pallidum_L	3	−19, −4, 2
	Thalamus_L	3	−19, −9, 5
Subtraction analysis ([apraxic group]-[pseudo-apraxic and unaffected groups])	Precentral_L	71	−42, 2, 31
	Frontal_Inf_Oper_L	71	−54, 14, 17
	Frontal_Inf_Tri_L	71	−50, 25, 17
	Rolandic_Oper_L	71	−38, −20, 20
	Insula_L	71	−37, −20, 20
	Parietal_Inf_L	71	−45, −25, 37
	Supra_Marginal_L	71	−46, −25, 35
	Frontal_Sup_L	57	−29, 26, 33
	Frontal_Mid_L	57	−33, 33, 17
	Postcentral_L	57	−48, −19, 25
	Parietal_Sup_L	57	−31, − 49, 51
	Angular_L	57	−40, −55, 22
	Temporal_Sup_L	57	−42, −40, 19
	Temporal_Mid_L	57	−42, −54, 22

*Overlapping regions of all patients and each group, with corresponding peak coordinates outlined in Montreal Neurological Institute (MNI) space, and the number of lesion overlaps are shown. Lesioned areas associated with apraxia with corresponding peak coordinates outlined in MNI space and the number of lesion overlaps after subtraction in percentage are shown. Only regions where there are many overlapping preferences have been reported. Angular_L, left angular gyrus; Frontal_Inf_Oper_L, left opercular part of the inferior frontal gyrus; Frontal_Inf_Tri_L, left triangular part of the inferior frontal gyrus; Frontal_Mid_L, left middle frontal gyrus; Frontal_Sup_L, left superior frontal gyrus; Insula_L, left insula; Pallidum_L, left pallidum; Parietal_Inf_L, left inferior parietal lobule; Parietal_Sup_L, left superior parietal lobule; Postcentral_L, left postcentral gyrus; Precentral_L, left precentral gyrus; Putamen_L, left putamen; Rolandic_Oper_L, left rolandic operculum; Supra_Marginal_L, left supramarginal gyrus; Temporal_Mid_L, left middle temporal gyrus; Temporal_Sup_L, left superior temporal gyrus; Thalamus_L, left thalamus*.

**Figure 6:**
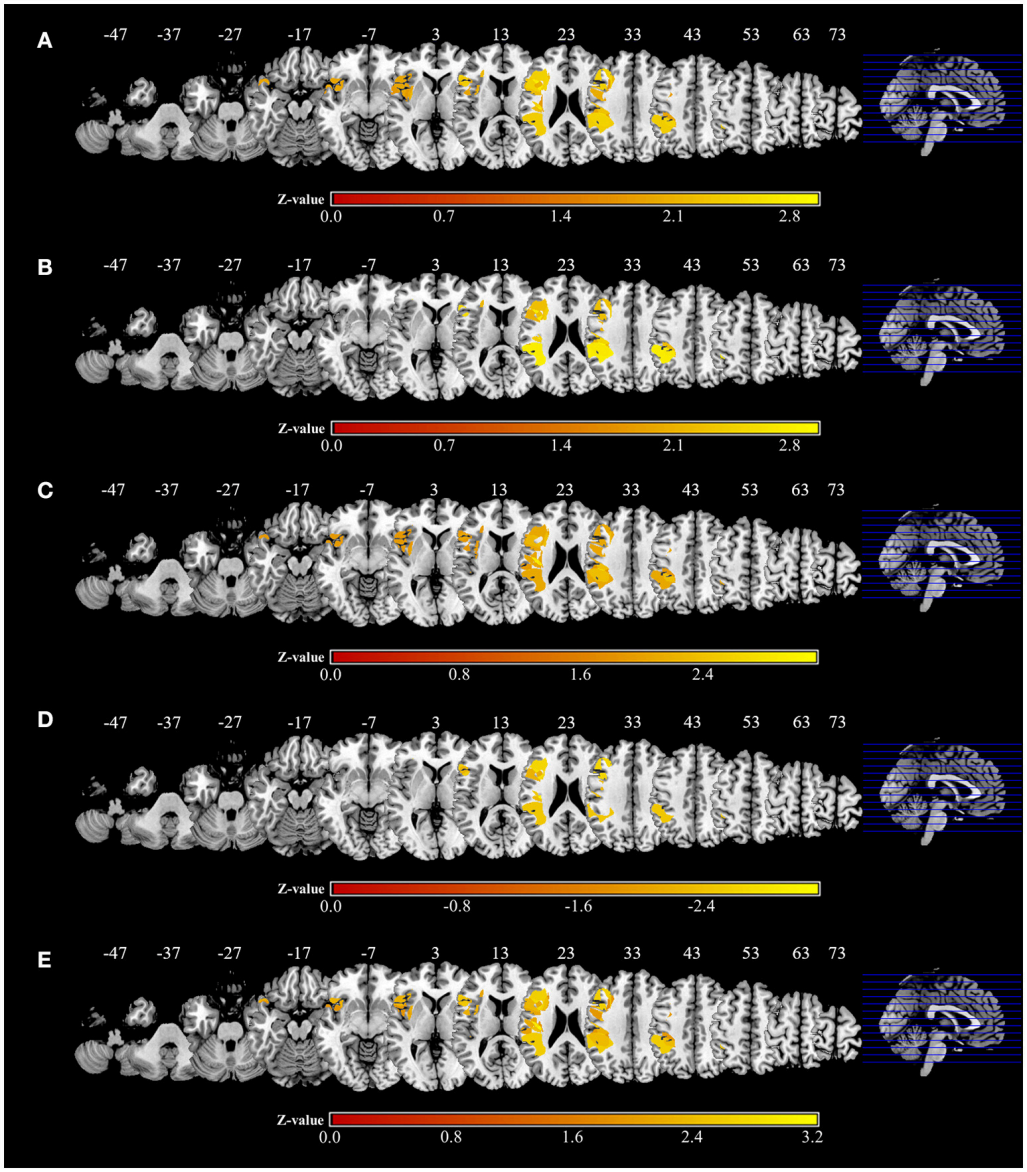
Voxel-based lesion-symptom mapping analysis. Color shades indicate z-scores (*z* = z-score). Axial slices with the Montreal Neurological Institute z coordinates from −47 to +73 are shown. Lesions significantly related to each factor are displayed in yellow. **(A)** Voxel-based lesion symptom mapping (VLSM) for the severity of apraxia (AST score). The results are shown at a false discovery rate (FDR)-corrected threshold of *p* < 0.05, *z* > 1.77. **(B)** VLSM for imitation deficits. FDR-corrected threshold of *p* < 0.05, *z* > 1.97. **(C)** VLSM for gesture deficits. FDR-corrected threshold of *p* < 0.05, *z* > 1.85. **(D)** VLSM for the delay detection threshold (DDT) for the detection of delayed visual feedback during active movements. FDR-corrected threshold of *p* < 0.05, *z* < −2.12. **(E)** VLSM for the steepness of the probability curve for the detection of delayed visual feedback during active movements. FDR-corrected threshold of *p* < 0.05, *z* > 1.82.

Significant associations of lesion location and task performance were assessed with VLSM (Figure 6, Table 3). For the severity of apraxia (deficits in the AST) (Figure 6A, Table 3), significant clusters of lesioned voxels were found within the left frontal cortex (precentral gyrus, superior frontal gyrus, middle frontal gyrus, IFG, including the opercular, triangular, and orbital regions, and rolandic operculum), left insula, and left parietal cortex (postcentral gyrus, superior parietal lobule, and IPL, including the supramarginal and angular gyrus). In addition, significant clusters of lesioned voxels were found within the left temporal cortex (superior temporal gyrus, superior temporal pole, and middle temporal gyrus). Subcortically, lesions affecting the left putamen were significantly associated with a lower accuracy in the AST (severe apraxia).

**Table 3:** Voxel-based lesion-symptom mapping (VLSM) showing associations between lesioned brain areas and the severity of apraxia (imitation and gesture deficits), and distortion of visuo-motor temporal integration.

**Factor**	**Lesion area**	**Number of lesioned voxels**	**Max/Min**	**X**	**Y**	**Z**
Apraxia severity	Precentral_L	28,174	2.742	−42	2	31
	Frontal_Sup_L	28,915	2.496	−29	25	33
	Frontal_Mid_L	38,722	2.496	−32	22	31
	Frontal_Inf_Oper_L	8,271	2.742	−54	14	17
	Frontal_Inf_Tri_L	20,104	2.742	−50	25	17
	Frontal_Inf_Orb_L	13,590	1.972	−43	19	−13
	Rolandic_Oper_L	7,939	2.953	−38	−20	20
	Insula_L	15,025	2.953	−37	−20	20
	Postcentral_L	31,053	2.468	−37	−35	41
	Parietal_Sup_L	16,519	2.397	−31	−49	51
	Parietal_Inf_L	19,447	2.737	−45	−25	37
	Supra_Marginal_L	9,907	2.737	−46	−25	35
	Angular_L	9,313	2.397	−40	−55	22
	Putamen_L	7,942	2.155	−33	−1	−4
	Temporal_Sup_L	18,307	2.397	−42	−40	19
	Temporal_Pole_Sup_L	10,228	1.972	−44	16	−17
	Temporal_Mid_L	39,353	2.397	−42	−54	22
Imitation deficits	Precentral_L	28,174	2.727	−42	2	31
	Frontal_Sup_L	28,915	2.424	−29	25	33
	Frontal_Mid_L	38,722	2.424	−32	22	31
	Frontal_Inf_Oper_L	8,271	2.727	−54	14	17
	Frontal_Inf_Tri_L	20,104	2.727	−50	25	17
	Rolandic_Oper_L	7,939	2.820	−38	−20	20
	Insula_L	15,025	2.938	−37	−20	24
	Postcentral_L	31,053	2.767	−43	−33	44
	Parietal_Sup_L	16,519	2.767	−31	−49	51
	Parietal_Inf_L	19,447	2.938	−44	−26	36
	Supra_Marginal_L	9,907	2.938	−46	−25	33
	Angular_L	9,313	2.767	−40	−55	22
	Temporal_Sup_L	18,307	2.767	−42	−40	19
	Temporal_Mid_L	39,353	2.767	−42	−54	22
Gesture deficits	Precentral_L	28,174	2.353	−46	−4	25
	Frontal_Sup_L	28,915	2.353	−29	25	33
	Frontal_Mid_L	38,722	2.353	−32	22	31
	Frontal_Inf_Oper_L	8,271	2.499	−55	15	18
	Frontal_Inf_Tri_L	20,104	2.499	−53	20	17
	Frontal_Inf_Orb_L	13,590	1.916	−43	19	−13
	Rolandic_Oper_L	7,939	3.130	−41	−17	24
	Insula_L	15,025	3.006	−40	−14	23
	Postcentral_L	31,053	2.916	−50	−8	19
	Parietal_Sup_L	16,519	2.086	−31	−49	51
	Parietal_Inf_L	19,447	2.762	−44	−26	36
	Supra_Marginal_L	9,907	2.762	−46	−25	33
	Angular_L	9,313	2.086	−40	−55	22
	Putamen_L	7,942	2.053	−33	−1	−4
	Temporal_Sup_L	18,307	2.353	−45	−32	19
	Temporal_Pole_Sup_L	10,228	1.916	−44	16	−17
	Temporal_Mid_L	39,353	2.086	−42	−54	22
DDT during active movements	Precentral_L	28,174	−2.770	−42	2	31
	Frontal_Sup_L	28,915	−2.648	−29	26	33
	Frontal_Mid_L	38,722	−2.648	−33	33	19
	Frontal_Inf_Oper_L	8,271	−2.770	−54	14	17
	Frontal_Inf_Tri_L	20,104	−2.770	−50	25	17
	Rolandic_Oper_L	7,939	−3.130	−38	−20	20
	Insula_L	15,025	−3.130	−37	−20	20
	Postcentral_L	31,053	−2.470	−49	−36	43
	Parietal_Sup_L	16,519	−2.465	−31	−49	51
	Parietal_Inf_L	19,447	−2.663	−45	−25	37
	Supra_Marginal_L	9,907	−2.663	−46	−25	35
	Angular_L	9,313	−2.465	−40	−55	22
	Temporal_Sup_L	18,307	−2.465	−42	−40	19
	Temporal_Mid_L	39,353	−2.465	−42	−54	22
Steepness during active movements	Precentral_L	28,174	2.788	−42	2	31
	Frontal_Sup_L	28,915	2.636	−29	26	33
	Frontal_Mid_L	38,722	2.636	−33	33	17
	Frontal_Inf_Oper_L	8,271	2.788	−54	14	17
	Frontal_Inf_Tri_L	20,104	2.788	−50	25	17
	Frontal_Inf_Orb_L	13,590	2.241	−39	19	−15
	Rolandic_Oper_L	7,939	3.205	−38	−20	20
	Insula_L	15,025	3.205	−37	−20	20
	Postcentral_L	31,053	2.925	−50	−8	19
	Parietal_Sup_L	16,519	2.595	−31	−49	51
	Parietal_Inf_L	19,447	2.804	−45	−25	37
	Supra_Marginal_L	9,907	2.804	−46	−25	35
	Angular_L	9,313	2.595	−40	−55	22
	Putamen_L	7,942	2.241	−33	0	−4
	Temporal_Sup_L	18,307	2.595	−42	−40	19
	Temporal_Pole_Sup_L	10,228	2.241	−55	17	−17
	Temporal_Mid_L	39,353	2.595	−42	−54	22

*For each region, the Montreal Neurological Institute coordinates of the center of mass are provided along with the maximum/minimum Brunner–Munzel (BM) z statistic obtained in each cluster and the number (n) of clustering voxels that survived the threshold of p < 0.05, false discovery rate (FDR)-corrected. DDT, the delay detection threshold for the detection of delayed visual feedback during active movements. Steepness, the steepness of the probability curve for the detection of delayed visual feedback during active movements. FDR-corrected threshold of VLSM for apraxia severity was p < 0.05, z > 1.77. FDR-corrected threshold of VLSM for imitation deficits was p < 0.05, z > 1.97. FDR-corrected threshold of VLSM for gesture deficits was p < 0.05, z > 1.85. FDR-corrected threshold of VLSM for DDT was p < 0.05, z < −2.12. FDR-corrected threshold of VLSM for steepness was p < 0.05, z > 1.82. Angular_L, left angular gyrus; Frontal_Inf_Oper_L, left opercular part of the inferior frontal gyrus; Frontal_Inf_Orb_L, left orbital part of inferior frontal gyrus; Frontal_Inf_Tri_L, left triangular part of the inferior frontal gyrus; Frontal_Mid_L, left middle frontal gyrus; Frontal_Sup_L, left superior frontal gyrus; Insula_L, left insula; Parietal_Inf_L, left inferior parietal lobule; Parietal_Sup_L, left superior parietal lobule; Postcentral_L, left postcentral gyrus; Precentral_L, left precentral gyrus; Putamen_L, left putamen; RolandicOper_L, left rolandic operculum; Insula_L, left insula; SupraMarginal_L, left supramarginal gyrus; Temporal_Mid_L, left middle temporal gyrus; TemporalPole_Sup_L, left superior temporal pole; Temporal_Sup_L, left superior temporal gyrus*.

The VLSM results based on imitation and gesture deficits are shown in Figures 6B,C, respectively (Table 3). The clusters of lesioned voxels significantly associated with gesture deficits were exactly the same as the VLSM results for apraxia severity (Figure 6C, Table 3). The clusters of lesioned voxels significantly associated with imitation deficits were similar to the lesions significantly associated with the severity of apraxia and gesture deficits, but did not include the orbital part of the IFG and temporal pole of the superior temporal gyrus (Figure 6B, Table 3).

As a result of VLSM for the DDT and steepness in the tactile and passive movement conditions, there were no significantly related lesioned voxels. However, in VLSM for the DDT and steepness in the active movement condition, there were significantly related lesioned voxels (Figures 6D,E, Table 3).

An extension of the DDT during active movements (deficits in visuo-motor temporal integration) (Figure 6D, Table 3) was significantly associated with lesioned voxels within the left frontal cortex (precentral gyrus, superior frontal gyrus, middle frontal gyrus, IFG, including the opercular and triangular regions, and rolandic operculum), left insula, and left parietal cortex (postcentral gyrus, superior parietal lobule, and IPL, including the supramarginal and angular gyrus). Furthermore, lesions of the left temporal cortex (superior temporal gyrus and middle temporal gyrus) were significantly associated with an increased DDT. Subcortically, there was no area significantly associated with DDT prolongation.

For decreases in steepness during active movements (deficits in visuo-motor temporal integration) (Figure 6E, Table 3), significant clusters of lesioned voxels were found within the left frontal cortex (precentral gyrus, superior frontal gyrus, middle frontal gyrus, IFG, including the opercular, triangular, and orbital regions, and rolandic operculum) and left insula as well as the left parietal cortex (postcentral gyrus, superior parietal lobule, and IPL, including the supramarginal and angular gyrus). Significant clusters of lesioned voxels were found within the left temporal cortex (superior temporal gyrus, superior temporal pole, and middle temporal gyrus). Subcortically, lesions affecting the left putamen were significantly associated with decreased steepness.

Finally, there was a significant correlation between lesion volume and the severity of apraxia (*r* = −0.893; *p* < 0.001), DDT (*r* = 0.716; *p* = 0.001), and steepness (*r* = −0.888; *p* < 0.001) in the active movement condition.

**Figure 7:**
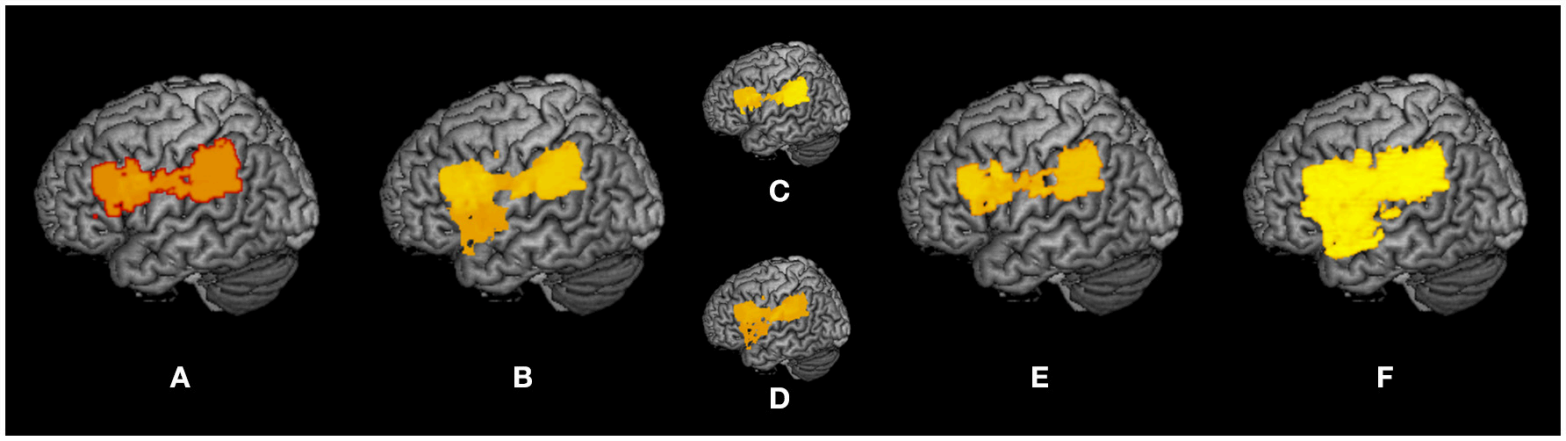
Summary of lesion analyses. Surface rendering reflects the projection of lesions onto the surface of the left hemisphere at any depth with maximum intensity. **(A)** Results of subtraction analyses of lesion overlap of patients in the pseudo-apraxic and unaffected groups from the lesion overlap of patients in the apraxic group. **(B)** Results of voxel-based lesion symptom mapping (VLSM) for the severity of apraxia. **(C)** Results of VLSM for imitation deficits. **(D)** Results of VLSM for gesture deficits. **(E)** Results of VLSM for the delay detection threshold (DDT) for the detection of delayed visual feedback during active movements. **(F)** Results of VLSM for the steepness of the probability curve for the detection of delayed visual feedback during active movements.

A summary of the subtraction and VLSM results is shown as a surface rendering in Figure 7 as a reflected lesion projection onto the surface of the left hemisphere. In the subtraction lesion analyses, they were included in the largest clusters (71%) of lesioned voxels (Figure 7A), and in the VLSM for the severity of apraxia (Figure 7B) (imitation deficits, Figure 7C; gesture deficits, Figure 7D), DDT extension (Figure 7E), and decrease of steepness (Figure 7F). All significantly related lesioned voxels were in the left IFG (including the opercular and triangular regions), left precentral gyrus, and left insula as well as the left IPL (including the supramarginal gyrus).

## Discussion

In this study, we evaluated the visuo-tactile, visuo-proprioceptive, and visuo-motor temporal integration functions of patients with apraxia by implementing three types (tactile stimulation and passive or active movement conditions) of delayed visual feedback detection tasks.

Our results showed that the DDT and steepness during active movements were extended and decreased, respectively, in the apraxic group compared to the unaffected and pseudo-apraxic groups. However, in the tactile stimulation and passive movement conditions, there was no difference in the DDT and steepness between the groups. Further, the DDT was extended significantly in the apraxic group during active movements compared to tactile stimulation and passive movements. These results indicated that apraxic patients had significantly distorted time windows of visuo-motor integration compared to non-apraxic patients and visuo-tactile/proprioceptive integration.

Correlation analyses showed that there was a significant correlation between the severity of apraxia and the indices of visuo-motor temporal integration (DDT and steepness). That is, as the difficulty of visuo-motor temporal integration increased, the severity of apraxia increased.

In addition, lesion analyses by subtraction and VLSM indicated that damage to the brain areas in the left ventro-dorsal stream, centered on the left IFG and left IPL, was significantly associated with both apraxia and distorted visuo-motor temporal integration.

### Specific distortion of visuo-motor temporal integration in limb apraxia

The current results suggested that apraxic patients had no difficulty in visuo-tactile and visuo-proprioceptive temporal integration, but had a specific difficulty with visuo-motor temporal integration. In this study, patients with a history of a mental disorder, developmental disability, cognitive disorder, impaired language comprehension precluding one's understanding of how to perform the experimental task, or impaired field of vision were excluded. In addition, this study included only patients with left hemispheric stroke, and the experimental tasks were conducted using the non-paralyzed left hand in the absence of sensory disturbance and a reduction in the sense of body ownership (asomatognosia and somatoparaphrenia). Furthermore, there were no significant differences between sex, age, educational background, general cognitive function, and length of time since stroke between the apraxic and non-apraxic (pseudo-apraxic and unaffected) patients. Therefore, the difficulty with visuo-motor temporal integration observed in patients with apraxia was not due to these factors.

Indeed, previous studies reported that visuo-motor integration in a novel motor learning task was difficult for patients with apraxia (44, 45). However, the current study evaluated the temporal aspects of visuo-motor integration. Therefore, the present study is the first to show that patients with apraxia had a deficit in visuo-motor temporal integration. That is, patients with apraxia had a distortion of the time window for integrating self-generated hand movements and visual information of their own hand. Here, we referred to the difficulty of visuo-motor temporal integration observed in apraxia as a distortion. In the active movement condition of the delayed visual feedback detection task, only the answer provided when a patient's left index finger flexion-extension movement was performed correctly was included in the analysis. Therefore, not only in non-apraxic limbs (pseudo-apraxic and unaffected) but also in apraxic limbs, the movement performed (flexion-extension of the left index finger) was correct. Specifically, in this study we observed a significant extension of the DDT and a significant decrease in steepness for visuo-motor temporal integration in apraxic limbs. When temporal errors between correctly executed self-generated movements and visual feedback were small, patients with apraxia could not detect the errors. Therefore, we defined the difficulty observed as a distortion of visuo-motor temporal integration.

The distortion of multisensory (including motor signals) temporal integration in apraxia was observed only in active movement conditions. The elements included in the visuo-motor temporal integration conditions (active movements), but not included in the visuo-tactile (tactile stimulation) and visuo-proprioceptive (passive movements) temporal integration conditions, were self-generated movements. Signals that arise during self-generated movements and not caused by integration between sensory feedback include motor prediction. Therefore, the difficulty with visuo-motor temporal integration observed in the apraxic group may be due to an impairment of motor prediction (e.g., efferent copy/predicted sensory feedback) during active movements (self-generated movements).

Several previous studies have suggested that a fundamental problem in apraxia is impaired motor prediction prior to the generation of a movement. In studies using motor imagery tasks (22, 87–90), patients with apraxia showed difficulty with mentally simulating movements of their own hands, and a significant correlation was noted between impaired motor imagery and impaired pantomiming of tool use (22). These previous results suggest that patients with apraxia have an impairment in anticipating the sensory consequences of hand movements (53). Two previous studies using electroencephalograms revealed that readiness potential, reflecting motor preparation, was missing in patients with apraxia (91, 92). This lack of readiness potential suggested that patients with apraxia cannot predict the consequences of their own motor actions (93, 94). A previous study by Wolpe et al. (95) used the “intentional binding” paradigm (the perceived temporal attraction between voluntary actions and their sensory effects) to study patients with corticobasal syndrome and apraxia. While there were no differences in the time taken to perceive sensory feedback, the apraxic limbs of patients with corticobasal syndrome had a significantly delayed perception of action (significantly increased “binding” of the time of actions to their effects) compared to unaffected limbs and the limbs of control subjects. In addition, there was a significant correlation between the binding effect and the severity of apraxia. This result suggested the reduced precision of voluntary action signals in patients with apraxia (95). These previous studies consistently showed that apraxia was associated with an impairment of motor prediction.

The current study did not use motor imagery tasks, electroencephalograms, or intentional binding paradigms. Instead, we compared visuo-tactile, visuo-proprioceptive, and visuo-motor temporal integration, and revealed that the time window for visuo-motor integration was distorted in patients with apraxia, while the time window for the integration of visuo-tactile and visuo-proprioceptive was preserved. Therefore, the previous studies and our data showed that the distortion of visuo-motor temporal integration in patients with apraxia was caused by deficits in motor prediction.

This explains the significant correlation between the severity of apraxia and distorted visuo-motor temporal integration (extended DDT and reduced steepness). Indeed, there is reportedly a significant correlation between the severity of apraxia and motor imagery impairments (22) and delayed perception of self-generated actions (95). Therefore, the present results, in agreement with previous studies (22, 95), suggested that the degree of deficit in motor prediction may affect the severity of apraxia. Previous studies have also shown that the detection of delayed visual feedback for active movements was better than the detection of delayed visual feedback for passive movements in healthy volunteers (58, 96). That is, the DDT and steepness were significantly shortened (96) and increased (58), respectively, in active movement conditions compared to passive movement conditions. The advantage of delay detection in an active movement condition is brought about by motor predictions associated with self-generated movements (58, 96). In the current study, delay detection of the unaffected group was significantly improved (increased steepness) in the active movement condition compared to the tactile stimuli condition. In contrast, delay detection of the apraxic group was significantly lower (extended DDT) in the active movement condition than in the tactile stimulation and passive movement conditions. This result strengthens the hypothesis that patients with apraxia show deficits of motor prediction.

### Common lesions between apraxia and the distortion of visuo-motor temporal integration

The subtraction and VLSM results showed that both the severity of apraxia of the left upper limb and the degree of distortion in visuo-motor temporal integration were significantly related to common lesions, e.g., in the IFG (including the opercular and triangular regions), left precentral gyrus, left rolandic operculum, left insula, and left IPL (including the supramarginal gyrus).

In the current study, the AST, which is a shortened version of the TULIA, was used to evaluate apraxia. Earlier VLSM studies investigating lesions associated with a low score (severe apraxia) for the TULIA also reported lesions such as in the left IFG (including the opercular, triangular, and orbital regions), left rolandic operculum, left insula, left pre-postcentral gyrus, left IPL (including the supramarginal and angular gyrus), and left superior temporal and superior temporal poles (97). The current results accurately matched those of these previous studies. Theta burst stimulation to the left IFG also reportedly significantly lowered the score for the TULIA (98). Furthermore, previous studies have also revealed that theta burst stimulation to the left IPL significantly reduced the imitation score in the TULIA, regardless of being meaningful or meaningless gestures (99). Therefore, the current results showing a significant association between the reduction (severe apraxia) of the AST score and lesions in the left IFG and IPL were consistent with the findings of previous stimulation studies. Previous lesion studies related to apraxia reported that lesions in the left IPL (21, 22, 69, 100–102), left IFG (39, 102, 103), left insula (104), and left precentral gyrus (102) were significantly associated with an impairment of transitive gestures (pantomime). These previous studies, in line with the current results, were performed using brain lesion areas associated with ipsilateral limb errors (apraxic symptoms). Previous imaging studies also reported that the left IPL (105–112), IFG (105, 106, 108, 111, 112), and left insula (106) were significantly associated with the execution of transitive gestures (pantomime). In addition, there were differences between hands and fingers, and between meaningful and meaningless gestures, but previous lesion studies reported that lesions significantly associated with imitation deficits were located in the left IPL (40, 49, 69, 101, 102, 113–116), left IFG (102, 113, 116), and left precentral gyrus (102). Therefore, in this study, the data regarding several lesions indicated by subtraction and VLSM for the severity of apraxia, i.e., in the left IFG (including the opercular and triangular regions), left precentral gyrus, left rolandic operculum, left insula, and left IPL (including the supramarginal gyrus), were consistent with previous lesion and imaging studies. This further indicated that the lesions of patients with apraxia who participated in the current study were general lesions that resulted in apraxia. The current study reconfirmed that the left IPL and left IFG in the left ventro-dorsal stream are important lesion regions for the development of apraxia, and the data were consistent with previous lesion and imaging studies.

The damaged areas related to imitation and gesture deficits were almost the same. However, the lesions associated with gesture deficits included lesions associated with imitation deficits, including slightly ventral areas (orbital part of the IFG and temporal pole of the superior temporal gyrus). Imitation is possible with simple visual-motor conversion, whereas gesture from verbal instruction requires semantic processing, and the ventral regions are more involved (100, 117). The current findings were consistent with the results of these previous studies and showed that the visuo-motor conversion region and semantic processing region overlap, but do not completely match.

In addition, the current lesion analysis revealed that these lesions were significantly related not only to the severity of apraxia but also to the degree of distortion (extended DDT and decreased steepness) in visuo-motor temporal integration. Activation of the IFG (including ventral premotor areas) has been reported in various functional imaging studies on finger movements (118–121), motor imagery of hand-fingers (122–124), motor learning of finger movements (125), and observation of finger movements. According to these previous investigations, a clear representation of finger movements exists in the IFG. The IPL is known to contribute to motor representations of hand and finger movements (126–129). Recently, the IFG was reported to have an important role in the visuo-motor integration of finger movements (130). The IFG and IPL are related to planning, execution, and feedback perception of finger movements, and also function in the visuo-motor integration of finger movements. Therefore, it is predicted that patients who have damage to these areas will have difficulty with visuo-motor integration. Damage to the left IFG (47, 116), left supramarginal gyrus, and left IPL (50, 85, 131, 132) impairs both the production and recognition of gestures. Therefore, the left IFG and left IPL are important areas for the production and recognition of hand movements (i.e., visuo-motor integration of hands). Consistent with these findings, our lesion analyses provided evidence that the left IFG and left IPL served as a time window for visuo-motor integration, but not visuo-tactile and visuo-proprioceptive functions.

Many studies have investigated brain regions involved in temporal errors between actions and feedback using delayed visual feedback, similar to the current study (133–144). Most of these studies have identified parietal areas that were active during such discrepancies, in particular the IPL (angular gyrus). Khalighinejad and Haggard (145) reported that anodal transcranial direct current stimulation to the left angular gyrus significantly reduced intentional binding regardless of whether the right or left hand was used. This effect was not observed after anodal stimulation to the right angular gyrus (145). Therefore, the left angular gyrus is suggested to have an important role in the integration of self-generated movements and perceptions. Another study (146) used functional MRI to measure a delayed visual feedback detection task for self-generated movements, similar to the current study, and found that the left angular gyrus was an important area for the detection of delay. The connectivity analysis in this study showed positive connectivity between the left angular gyrus and left frontal regions. This previous study concluded that the left angular gyrus was a supramodal comparator area in action-outcome monitoring, as significant activity was observed in the left angular gyrus not only during visual feedback but also during the detection of delayed auditory feedback, further suggesting that the left angular gyrus and connected frontal processes are specifically relevant for the comparison of predicted and perceived time points of the consequences of visual actions (146). Straube et al. (147) showed that transcranial direct current anodal stimulation to the left frontal-parietal regions (left dorsolateral prefrontal cortex and left angular gyrus) significantly improved the detection of delayed visual feedback for self-initiated movements compared to cathodal stimulation. In contrast, cathodal stimulation to the left frontal-parietal (and anodal stimulation to the right frontal-parietal) provided a benefit to the detection of delay in visual feedback for passive movements. This previous study concluded that the frontal-parietal network of the left hemisphere has a more important role than the right hemisphere in the generation of motor predictions and for the comparison of predicted and perceived time points of sensory information (147). These previous studies provided evidence that directly supports the current results. Our results showed that damage to the left inferior fronto-parietal (including the left middle frontal gyrus and angular gyrus) did not affect delay detection in visual feedback for tactile stimuli and passive movements, but had a negative effect on the delayed detection of visual feedback for self-generated movements. Thus, consistent with these previous studies, the current study provided additional evidence for the significance of the left hemisphere in temporal comparison/integration between motor prediction and actual feedback.

The VLSM results in the current study showed that damage to the insula is involved in both apraxia and the distortion of visuo-motor temporal integration. Lesions in the insula have been reported to cause apraxia (104, 106). Conversely, the insula receives various inputs and has various functions such as error awareness, moment of recognition, decision making, time perception, self-recognition, interoception, and rhythm (148). Furthermore, previous studies revealed that the insula is involved not only in the generation of buccofacial and limb actions but also in the recognition of action sounds (46, 47). Therefore, damage to the insula does not specifically cause only apraxia or distortion of visuo-motor temporal integration.

Apraxia is thought to be caused by an impairment of “stored motor representation” (19–22, 31, 52, 149–152), “technical reasoning” (mechanical problem solving) (153–165), and/or “body part coding” (113, 166–168) functioning in the regions (especially the left IPL) of the left ventro-dorsal stream. Here, it is important to emphasize that the present study does not insist that the impairment of stored motor representation, technical reasoning, or body part coding is not a cause of apraxia. There is evidence showing that the IFG and IPL in the left ventro-dorsal stream are engaged in the mental simulation of hand-finger movements (169–171), motor learning (generation of a forward model) based on a comparison of motor prediction and actual feedback (172–174), production and recognition of gestures (47, 50, 85, 116, 131, 132), and visuo-motor integration (130), in addition to functions that are the main causes of apraxia. These findings suggested that in the left ventro-dorsal stream, the basic functions of the temporal integration of self-generated movements and visual feedback are carried out downstream of the main function, which causes apraxia. Therefore, the current findings indicated that damage to the inferior frontal-parietal regions in the left ventro-dorsal stream caused not only apraxia but also a distortion of the time window of visuo-motor integration operating downstream of the mechanism underlying apraxia.

In addition, cognitive models for apraxia predict that deficits of visuo-motor conversion result in deficits of meaningless gesture imitations (conduction apraxia) (35). However, the current results showed that the distortion of visuo-motor temporal integration is related to various symptoms of apraxia, e.g., not only imitation deficits of meaningless intransitive gestures but also imitation deficits of meaningful intransitive gestures, imitation deficits of pantomimes, deficits of intransitive gestures from verbal instructions, and deficits of pantomimes from verbal instructions.

Finally, the present study revealed that there are deficits in visuo-motor temporal integration in patients with apraxia, although visuo-tactile and visuo-proprioceptive temporal integration are preserved. Therefore, it may be possible to help visuo-motor integration, promote motor prediction, and improve the symptoms of apraxia through normal visual, tactile, proprioceptive, and other sensations. Assistive technology devices (175–177), and virtual and augmented reality neurorehabilitation (178, 179) may be useful in this regard (180). In addition, action observation therapy (181) and motor imagery training (182) may be effective in promoting visuo-motor integration and improving the symptoms of apraxia (180). Therefore, future clinical trials are needed to verify whether these approaches ameliorate these symptoms.

### Limitations of the current study and future directions

The current study has several limitations that must be noted. Only the MMSE was used to evaluate the cognitive function of the patients. Therefore, the state of other cognitive functions may have influenced the results of the experimental task. Thus, future studies need to investigate cognitive function in more detail.

There was a large range of disease duration in the current study; therefore, the effects of neuroplasticity and adaptation might have influenced the results. We found a significant correlation between lesion volume and the severity of apraxia (AST score) and experimental variables (DDT and steepness in the active movement condition). Furthermore, the sample size in this study was very small, and the slice thickness (6 mm) of MRI used in lesion analyses was coarse. Therefore, the current study could not sufficiently clarify the differences in lesions related to apraxia and the distortion of visuo-motor temporal integration. Therefore, future studies that match disease duration and lesion size, increase sample size, and improve the imaging technique to clarify the difference between apraxia-causing lesions and visuo-motor temporal integration distortion-causing lesions are necessary.

In addition, the current study was unable to investigate in detail the relationship between various apraxia symptoms and distortions of visuo-motor temporal integration and lesions, according to the cognitive models for apraxia. In conformity with the cognitive models for apraxia, future studies are needed to investigate the classification of apraxia symptoms and the corresponding lesions. Future studies may be better to use the test battery by Bartolo et al. (34), which enables the evaluation of detailed apraxic symptoms based on the cognitive models.

The current results do not insist that apraxia is mainly caused by a distortion of visuo-motor temporal integration. We did not investigate the relationship between symptoms other than apraxia and the time window of multisensory integration. The superior parietal lobule of the dorso-dorsal stream is known to make an important contribution to visuo-motor integration (183–185). Optic ataxia is a typical visuo-motor disorder caused by damage in these areas (186–188). Therefore, a distortion of visuo-motor temporal integration may be observed in other brain lesions (especially those in the superior parietal lobule) and with other symptoms (especially optic ataxia, which is a typical visuo-motor disorder). Further studies investigating the time window of multisensory integration (including motor prediction) for other brain lesions or symptoms, using a paradigm similar to that of the present study, will contribute to our understanding of the brain mechanism underlying multisensory integration and symptoms.

## Conclusions

Patients with apraxia have specific distortions of visuo-motor temporal integration, but the temporal integration of visuo-tactile and visuo-proprioceptive information is preserved. The degree of distortion of visuo-motor temporal integration is significantly correlated with the severity of apraxia. Damage to the left IFG and left IPL in the left ventro-dorsal stream is a common lesion significantly related to apraxia and the distortion of visuo-motor temporal integration. However, in order to understand apraxia more deeply, future studies that take into account several of the limitations of the present study are necessary.

## Ethics statement

The experimental procedures were approved by the local ethics committee of the Graduate School and Faculty of Health Sciences at Kio University (approval number: H27-16). There were no foreseeable risks to the participants, and no personally identifying information was collected. The participants provided background information and written informed consent. The procedures complied with the ethical standards of the 1964 Declaration of Helsinki regarding the treatment of human participants in research.

## Author contributions

SN collected and analyzed the data and wrote the manuscript. RI and YTak manually delineated the boundaries of the ischemic lesions in the lesion analyses. YTak assisted in the lesion analyses. EO, YTan, MK, TT, YI, HO, and KN assisted in collecting the data. SN, TZ, and MO designed the study. SS and SM designed and supervised the study. All authors read and approved the manuscript.

### Conflict of interest statement

The authors declare that the research was conducted in the absence of any commercial or financial relationships that could be construed as a potential conflict of interest. The reviewer NDL and handling Editor declared their shared affiliation.

## References

[1] Blakemore SJ Sirigu A. Action prediction in the cerebellum and in the parietal lobe. Exp Brain Res. (2003) 153:239–45. 10.1007/s00221-003-1597-z 12955381

[2] Davidson PR Wolpert DM. Widespread access to predictive models in the motor system: a short review. J Neural Eng. (2005) 2:S313–319. 10.1088/1741-2560/2/3/S11 16135891

[3] Wolpert DM Ghahramani Z Jordan MI. An internal model for sensorimotor integration. Science (1995) 269:1880–2. 10.1126/science.7569931 7569931

[4] Kawato M. Internal models for motor control and trajectory planning. Curr Opin Neurobiol. (1999) 9:718–27. 10.1016/S0959-4388(99)00028-8 10607637

[5] Miall RC Weir DJ Wolpert DM Stein JF. Is the cerebellum a smith predictor? J Mot Behav. (1993) 25:203–16. 10.1080/00222895.1993.9942050 12581990

[6] Wolpert DM. Computational approaches to motor control. Trends Cogn Sci. (1997) 1:209–16. 10.1016/S1364-6613(97)01070-X 21223909

[7] Shadmehr R Smith MA Krakauer JW. Error correction, sensory prediction, and adaptation in motor control. Annu Rev Neurosci. (2010) 33:89–108. 10.1146/annurev-neuro-060909-153135 20367317

[8] Hyde C Wilson PH. Dissecting online control in Developmental Coordination Disorder: a kinematic analysis of double-step reaching. Brain Cogn. (2011) 75:232–41. 10.1016/j.bandc.2010.12.004 21256656

[9] Hyde C Wilson P. Online motor control in children with developmental coordination disorder: chronometric analysis of double-step reaching performance. Child Care Health Dev. (2011) 37:111–22. 10.1111/j.1365-2214.2010.01131.x 20637020

[10] Wolpert DM Goodbody SJ Husain M. Maintaining internal representations: the role of the human superior parietal lobe. Nat Neurosci. (1998) 1:529–33. 10.1038/2245 10196553

[11] Wolpert DM Miall RC Kawato M. Internal models in the cerebellum. Trends Cogn Sci. (1998) 2:338–47. 10.1016/S1364-6613(98)01221-2 21227230

[12] Desmurget M Epstein CM Turner RS Prablanc C Alexander GE Grafton ST. Role of the posterior parietal cortex in updating reaching movements to a visual target. Nat Neurosci. (1999) 2:563–7. 10.1038/9219 10448222

[13] Desmurget M Grafton S. Forward modeling allows feedback control for fast reaching movements. Trends Cogn Sci. (2000) 4:423–31. 10.1016/S1364-6613(00)01537-0 11058820

[14] Todorov E Jordan MI. Optimal feedback control as a theory of motor coordination. Nat Neurosci. (2002) 5:1226–35. 10.1038/nn963 12404008

[15] Tseng YW Diedrichsen J Krakauer JW Shadmehr R Bastian AJ. Sensory prediction errors drive cerebellum-dependent adaptation of reaching. J Neurophysiol. (2007) 98:54–62. 10.1152/jn.00266.2007 17507504

[16] Shadmehr R Krakauer JW. A computational neuroanatomy for motor control. Exp Brain Res. (2008) 185:359–81. 10.1007/s00221-008-1280-5 18251019 PMC2553854

[17] Heilman KM Rothi LJ Valenstein E. Two forms of ideomotor apraxia. Neurology (1982) 32:342–6. 10.1212/WNL.32.4.342 7199656

[18] Rothi LJG Ochipa C Heilman KM A Cognitive Neuropsychological Model of Limb Praxis. Cogn Neuropsychol. (1991) 8:443–58. 10.1080/02643299108253382

[19] Buxbaum LJ Veramonti T Schwartz MF Function and manipulation tool knowledge in apraxia: knowing ‘what for’ but not ‘how’. Neurocase (2000) 6:83–97. 10.1080/13554790008402763

[20] Buxbaum LJ Saffran EM. Knowledge of object manipulation and object function: dissociations in apraxic and nonapraxic subjects. Brain Lang. (2002) 82:179–99. 10.1016/S0093-934X(02)00014-7 12096875

[21] Buxbaum LJ Sirigu A Schwartz MF Klatzky R. Cognitive representations of hand posture in ideomotor apraxia. Neuropsychologia (2003) 41:1091–113. 10.1016/S0028-3932(02)00314-7 12667544

[22] Buxbaum LJ Johnson-Frey SH Bartlett-Williams M. Deficient internal models for planning hand-object interactions in apraxia. Neuropsychologia (2005) 43:917–29. 10.1016/j.neuropsychologia.2004.09.006 15716162

[23] Kellenbach M Brett M Patterson K. Actions speak louder than functions: the importance of manipulability and action in tool representation. J Cogn Neurosci. (2003) 15:30–46. 10.1162/089892903321107800 12590841

[24] Boronat C Buxbaum L Coslett H Tang K Saffran EM Kimberg DY. Distinctions between manipulation and function knowledge of objects: evidence from functional magnetic resonance imaging. Cogn Brain Res. (2005) 23:361–73. 10.1016/j.cogbrainres.2004.11.001 15820643

[25] Canessa N Borgo F Cappa SF Perani D Falini A Buccino G. The different neural correlates of action and functional knowledge in semantic memory: an FMRI study. Cereb Cortex (2008) 18:740–51. 10.1093/cercor/bhm110 17621607

[26] Rueschemeyer SA van Rooij D Lindemann O Willems RM Bekkering H. The function of words: distinct neural correlates for words denoting differently manipulable objects. J Cogn Neurosci. (2010) 22:1844–51. 10.1162/jocn.2009.21310 19583471

[27] Evans C Edwards MG Taylor LJ Ietswaart M. Perceptual decisions regarding object manipulation are selectively impaired in apraxia or when tDCS is applied over the left IPL. Neuropsychologia (2016) 86:153–66. 10.1016/j.neuropsychologia.2016.04.020 27109034

[28] Rizzolatti G Matelli M. Two different streams form the dorsal visual system: anatomy and functions. Exp Brain Res. (2003) 153:146–57. 10.1007/s00221-003-1588-0 14610633

[29] Frey SH. Tool use, communicative gesture and cerebral asymmetries in the modern human brain. Philos Trans R Soc Lond B Biol Sci. (2008) 363:1951–7. 10.1098/rstb.2008.0008 18292060 PMC2606701

[30] Króliczak G Frey SH. A common network in the left cerebral hemisphere represents planning of tool use pantomimes and familiar intransitive gestures at the hand-independent level. Cereb Cortex (2009) 19:2396–410. 10.1093/cercor/bhn261 19181695 PMC2742597

[31] Buxbaum LJ Kalénine S. Action knowledge, visuomotor activation, and embodiment in the two action systems. Ann NY Acad Sci. (2010) 1191:201–18. 10.1111/j.1749-6632.2010.05447.x 20392282 PMC4311774

[32] Cubelli R. Definition: apraxia. Cortex (2017) 93:227. 10.1016/j.cortex.2017.03.012 28410624

[33] Bartolo A Cubelli R. The cognitive models of limb apraxia and the specific properties of meaningful gestures. Cortex (2014) 57:297–8; discussion 306-308. 10.1016/j.cortex.2014.01.007 24559771

[34] Bartolo A Cubelli R Della Sala S. Cognitive approach to the assessment of limb apraxia. Clin Neuropsychol. (2008) 22:27–45. 10.1080/13854040601139310 17853141

[35] Cubelli R Marchetti C Boscolo G Della Sala S. Cognition in action: testing a model of limb apraxia. Brain Cogn. (2000) 44:144–65. 10.1006/brcg.2000.1226 11041987

[36] Buxbaum LJ Coslett HB Spatiomotor aspects of action. In: Rapp B, editor. The Handbook of Cognitive Neuropsychology: What Deficits Reveal About the Human Mind. New York, NY: Taylor & Francis Group; Psychology Press (2001). pp. 543–63.

[37] Buxbaum LJ Kyle KM Menon R. On beyond mirror neurons: internal representations subserving imitation and recognition of skilled object-related actions in humans. Brain Res Cogn Brain Res. (2005) 25:226–39. 10.1016/j.cogbrainres.2005.05.014 15996857

[38] Buxbaum LJ Kyle K Grossman M Coslett HB. Left inferior parietal representations for skilled hand-object interactions: evidence from stroke and corticobasal degeneration. Cortex (2007) 43:411–23. 10.1016/S0010-9452(08)70466-0 17533764

[39] Goldenberg G Hermsdörfer J Glindemann R Rorden C Karnath HO. Pantomime of tool use depends on integrity of left inferior frontal cortex. Cereb Cortex (2007) 17:2769–76. 10.1093/cercor/bhm004 17339607

[40] Haaland KY Harrington DL Knight RT. Neural representations of skilled movement. Brain (2000) 123:2306–13. 10.1093/brain/123.11.2306 11050030

[41] Halsband U Schmitt J Weyers M Binkofski F Grützner G Freund HJ. Recognition and imitation of pantomimed motor acts after unilateral parietal and premotor lesions: a perspective on apraxia. Neuropsychologia (2001) 39:200–16. 10.1016/S0028-3932(00)00088-9 11163376

[42] Heilman KM Schwartz HD Geschwind N. Defective motor learning in ideomotor apraxia. Neurology (1975) 25:1018–20. 10.1212/WNL.25.11.1018 1237817

[43] Motomura N Seo T Asaba H Sakai T. Motor learning in ideomotor apraxia. Int J Neurosci. (1989) 47:125–9. 10.3109/00207458908987424 2793336

[44] Mutha PK Sainburg RL Haaland KY. Coordination deficits in ideomotor apraxia during visually targeted reaching reflect impaired visuomotor transformations. Neuropsychologia (2010) 48:3855–67. 10.1016/j.neuropsychologia.2010.09.018 20875439 PMC3712783

[45] Mutha PK Stapp LH Sainburg RL Haaland KY. Motor Adaptation Deficits in Ideomotor Apraxia. J Int Neuropsychol Soc. (2017) 23:139–49. 10.1017/S135561771600120X 28205499 PMC5374977

[46] Pazzaglia M Pizzamiglio L Pes E Aglioti SM. The sound of actions in apraxia. Curr Biol. (2008) 18:1766–72. 10.1016/j.cub.2008.09.061 19013068

[47] Pazzaglia M Smania N Corato E Aglioti SM. Neural underpinnings of gesture discrimination in patients with limb apraxia. J Neurosci. (2008) 28:3030–41. 10.1523/JNEUROSCI.5748-07.2008 18354006 PMC6670701

[48] Rothi LJG Heilman KM. Acquisition and retention of gestures by apraxic patients. Brain Cogn. (1984) 3:426–37. 10.1016/0278-2626(84)90032-0 6085679

[49] Weiss PH Dohle C Binkofski F Schnitzler A Freund HJ Hefter H. Motor impairment in patients with parietal lesions: disturbances of meaningless arm movement sequences. Neuropsychologia (2001) 39:397–405. 10.1016/S0028-3932(00)00129-9 11164878

[50] Weiss PH Rahbari NN Hesse MD Fink GR. Deficient sequencing of pantomimes in apraxia. Neurology (2008) 70:834–40. 10.1212/01.wnl.0000297513.78593.dc 18332341

[51] Heilman KM Rothi LJG Apraxia. In: Heilman KM Valenstein E, editors. Clinical neuropsychology. New York, NY: Oxford University Press (1993). pp. 141–64.

[52] Buxbaum LJ. Moving the gesture engram into the 21st century. Cortex (2014) 57:286–9; discussion 306–308. 10.1016/j.cortex.2014.01.006 24552694 PMC4108537

[53] Pazzaglia M Galli G. Loss of agency in apraxia. Front Hum Neurosci. (2014) 8:751. 10.3389/fnhum.2014.00751 25295000 PMC4172088

[54] Jaime M Longard J Moore C. Developmental changes in the visual-proprioceptive integration threshold of children. J Exp Child Psychol. (2014) 125:1–12. 10.1016/j.jecp.2013.11.004 24814203

[55] Nobusako S Sakai A Tsujimoto T Shuto T Nishi Y Asano D. Deficits in visuo-motor temporal integration impacts manual dexterity in probable developmental coordination disorder. Front Neurol. 9:114. 10.3389/fneur.2018.00114.eCollection2018 29556211 PMC5844924

[56] Nobusako S Sakai A Tsujimoto T Shuto T Nishi Y Asano D. Manual dexterity is a strong predictor of visuo-motor temporal integration in children. Front Psychol. (2018) 9:948. 10.3389/fpsyg.2018.00948 29946283 PMC6005835

[57] Shimada S Fukuda K Hiraki K. Rubber hand illusion under delayed visual feedback. PLoS ONE (2009) 4:e6185. 10.1371/journal.pone.0006185 19587780 PMC2702687

[58] Shimada S Qi Y Hiraki K. Detection of visual feedback delay in active and passive self-body movements. Exp Brain Res. (2010) 201:359–64. 10.1007/s00221-009-2028-6 19830411

[59] Shimada S Suzuki T Yoda N Hayashi T. Relationship between sensitivity to visuotactile temporal discrepancy and the rubber hand illusion. Neurosci Res. (2014) 85:33–8. 10.1016/j.neures.2014.04.009 24874005

[60] Garbarini F Rabuffetti M Piedimonte A Pia L Ferrarin M Frassinetti F. ‘Moving’ a paralysed hand: bimanual coupling effect in patients with anosognosia for hemiplegia. Brain (2012) 135(Pt 5):1486–97. 10.1093/brain/aws015 22374937

[61] Garbarini F Fossataro C Berti A Gindri P Romano D Pia L. When your arm becomes mine: pathological embodiment of alien limbs using tools modulates own body representation. Neuropsychologia (2015) 70:402–13. 10.1016/j.neuropsychologia.2014.11.008 25448852

[62] Pia L Garbarini F Fossataro C Fornia L Berti A. Pain and body awareness: evidence from brain-damaged patients with delusional body ownership. Front Hum Neurosci. (2013) 7:298. 10.3389/fnhum.2013.00298 23801958 PMC3687253

[63] Pia L Spinazzola L Rabuffetti M Ferrarin M Garbarini F Piedimonte A. Temporal coupling due to illusory movements in bimanual actions: evidence from anosognosia for hemiplegia. Cortex (2013) 49:1694–703. 10.1016/j.cortex.2012.08.017 23021071

[64] Ozkan S Adapinar DO Elmaci NT Arslantas D. Apraxia for differentiating Alzheimer's disease from subcortical vascular dementia and mild cognitive impairment. Neuropsychiatr Dis Treat. (2013) 9:947–51. 10.2147/NDT.S47879 23882142 PMC3709829

[65] Smits LL Flapper M Sistermans N Pijnenburg YA Scheltens P van der Flier WM. Apraxia in mild cognitive impairment and Alzheimer's disease: validity and reliability of the Van Heugten test for apraxia. Dement Geriatr Cogn Disord. (2014) 38:55–64. 10.1159/000358168 24603451

[66] Maeda T Kato M Muramatsu T Iwashita S Mimura M Kashima H. Aberrant sense of agency in patients with schizophrenia: forward and backward over-attribution of temporal causality during intentional action. Psychiatry Res. (2012) 198:1–6. 10.1016/j.psychres.2011.10.021 22374553

[67] Maeda T Takahata K Muramatsu T Okimura T Koreki A Iwashita S. Reduced sense of agency in chronic schizophrenia with predominant negative symptoms. Psychiatry Res. (2013) 209:386–92. 10.1016/j.psychres.2013.04.017 23680465

[68] Beker S Foxe JJ Molholm S. Ripe for solution: delayed development of multisensory processing in autism and its remediation. Neurosci Biobehav Rev. (2018) 84:182–92. 10.1016/j.neubiorev.2017.11.008 29162518 PMC6389331

[69] Goldenberg G Randerath J. Shared neural substrates of apraxia and aphasia. Neuropsychologia (2015) 75:40–9. 10.1016/j.neuropsychologia.2015.05.017 26004063

[70] Japan Society for Higher Brain Dysfunction The Standard Language Test of Aphasia (SLTA). Tokyo: Shinkoh-Igaku-Shuppansha (2003).

[71] Japan Society for Higher Brain Dysfunction The Supplementary Tests for Standard Language Test of Aphasia (SLTA-ST). Tokyo: Shinkoh-Igaku-Shuppansha (2011).

[72] Oldfield RC. The assessment and analysis of handedness: the Edinburgh inventory. Neuropsychologia (1971) 9:97–113. 10.1016/0028-3932(71)90067-4 5146491

[73] Liu M Chino N Tuji T Masakado Y Hase K Kimura A. Psychometric properties of the stroke impairment assessment set (SIAS). Neurorehabil Neural Repair. (2002) 16:339–51. 10.1177/0888439002239279 12462765

[74] Feinberg TE Haber LD Leeds NE. Verbal asomatognosia. Neurology (1990) 40:1391–4. 10.1212/WNL.40.9.1391 2392224

[75] Vanbellingen T Kersten B Van Hemelrijk B Van de Winckel A Bertschi M Müri R. Comprehensive assessment of gesture production: a new test of upper limb apraxia (TULIA). Eur JNeurol. (2010) 17:59–66. 10.1111/j.1468-1331.2009.02741.x 19614961

[76] Vanbellingen T Kersten B Van de Winckel A Bellion M Baronti F Müri R. A new bedside test of gestures in stroke: the apraxia screen of TULIA (AST). J Neurol Neurosurg Psychiatry (2011) 82:389–92. 10.1136/jnnp.2010.213371 20935324

[77] Dovern A Fink GR Weiss PH. Diagnosis and treatment of upper limb apraxia. J Neurol. (2012) 259:1269–83. 10.1007/s00415-011-6336-y 22215235 PMC3390701

[78] Afraz SR Kiani R Esteky H. Microstimulation of inferotemporal cortex influences face categorization. Nature (2006) 442:692–5. 10.1038/nature04982 16878143

[79] Rorden C Karnath HO Bonilha L. Improving lesion-symptom mapping. J Cogn Neurosci. (2007) 19:1081–8. 10.1162/jocn.2007.19.7.1081 17583985

[80] Rorden C Bonilha L Fridriksson J Bender B Karnath HO. Age-specific CT and MRI templates for spatial normalization. Neuroimage (2012) 61:957–65. 10.1016/j.neuroimage.2012.03.020 22440645 PMC3376197

[81] Ashburner J Friston KJ. Unified segmentation. Neuroimage (2005) 26:839–51. 10.1016/j.neuroimage.2005.02.018 15955494

[82] Winder K Seifert F Köhrmann M Crodel C Kloska S Dörfler A. Lesion mapping of stroke-related erectile dysfunction. Brain (2017) 140:1706–17. 10.1093/brain/awx080 28430885

[83] Seifert CL Schönbach EM Magon S Gross E Zimmer C Förschler A. Headache in acute ischaemic stroke: a lesion mapping study. Brain (2016) 139:217–26. 10.1093/brain/awv333 26603369

[84] Timpert DC Weiss PH Vossel S Dovern A Fink GR. Apraxia and spatial inattention dissociate in left hemisphere stroke. Cortex (2015) 71:349–58. 10.1016/j.cortex.2015.07.023 26298504

[85] Martin M Dressing A Bormann T Schmidt CSM Kümmerer D Beume L. Componential network for the recognition of tool-associated actions: evidence from voxel-based lesion-symptom mapping in acute stroke patients. Cereb Cortex (2017) 27:4139–52. 10.1093/cercor/bhw226 27497285

[86] Tzourio-Mazoyer N Landeau B Papathanassiou D Crivello F Etard O Delcroix N. Automated anatomical labeling of activations in SPM using a macroscopic anatomical parcellation of the MNI MRI single-subject brain. Neuroimage (2002) 15:273–89. 10.1006/nimg.2001.0978 11771995

[87] Sirigu A Duhamel JR Cohen L Pillon B Dubois B Agid Y. The mental representation of hand movements after parietal cortex damage. Science (1996) 273:1564–8. 10.1126/science.273.5281.1564 8703221

[88] Sirigu A Duhamel JR. Motor and visual imagery as two complementary but neurally dissociable mental processes. J Cogn Neurosci. (2001) 13:910–9. 10.1162/089892901753165827 11595094

[89] Ochipa C Rapcsak SZ Maher LM Rothi LJ Bowers D Heilman KM. Selective deficit of praxis imagery in ideomotor apraxia. Neurology (1997) 49:474–80. 10.1212/WNL.49.2.474 9270580

[90] Tomasino B Rumiati RI Umiltà CA. Selective deficit of motor imagery as tapped by a left-right decision of visually presented hands. Brain Cogn. (2003) 53:376–80. 10.1016/S0278-2626(03)00147-7 14607185

[91] Sirigu A Daprati E Ciancia S Giraux P Nighoghossian N Posada A. Altered awareness of voluntary action after damage to the parietal cortex. Nat Neurosci. (2004) 7:80–4. 10.1038/nn1160 14647290

[92] Fontana AP Kilner JM Rodrigues EC Joffily M Nighoghossian N Vargas CD. Role of the parietal cortex in predicting incoming actions. Neuroimage (2012) 59:556–64. 10.1016/j.neuroimage.2011.07.046 21839178

[93] Pazzaglia M. Does what you hear predict what you will do and say? Behav Brain Sci. (2013) 36:370–1. 10.1017/S0140525X12002804 23789973

[94] Pazzaglia M. Impact commentaries. Action discrimination: impact of apraxia. J Neurol Neurosurg Psychiatry (2013) 84:477–8. 10.1136/jnnp-2012-304817 23463866

[95] Wolpe N Moore JW Rae CL Rittman T Altena E Haggard P. The medial frontal-prefrontal network for altered awareness and control of action in corticobasal syndrome. Brain (2014) 137:208–20. 10.1093/brain/awt302 24293266 PMC3891444

[96] van Kemenade BM Arikan BE Kircher T Straube B. Predicting the sensory consequences of one's own action: first evidence for multisensory facilitation. Atten Percept Psychophys. (2016) 78:2515–26. 10.3758/s13414-016-1189-1 27515031

[97] Candidi M Sacheli LM Era V Canzano L Tieri G Aglioti SM. Come together: human-avatar on-line interactions boost joint-action performance in apraxic patients. Soc Cogn Affect Neurosci. (2017) 12:1793–802. 10.1093/scan/nsx114 29140533 PMC5714226

[98] Bohlhalter S Vanbellingen T Bertschi M Wurtz P Cazzoli D Nyffeler T. Interference with gesture production by theta burst stimulation over left inferior frontal cortex. Clin Neurophysiol. (2011) 122:1197–202. 10.1016/j.clinph.2010.11.008 21130031

[99] Vanbellingen T Bertschi M Nyffeler T Cazzoli D Wiest R Bassetti C. Left posterior parietal theta burst stimulation affects gestural imitation regardless of semantic content. Clin Neurophysiol. (2014) 125:457–62. 10.1016/j.clinph.2013.07.024 24054467

[100] Hoeren M Kümmerer D Bormann T Beume L Ludwig VM Vry MS. Neural bases of imitation and pantomime in acute stroke patients: distinct streams for praxis. Brain (2014) 137(Pt 10):2796–810. 10.1093/brain/awu203 25062694

[101] Martin M Nitschke K Beume L Dressing A Bühler LE Ludwig VM. Brain activity underlying tool-related and imitative skills after major left hemisphere stroke. Brain (2016) 139(Pt 5):1497–516. 10.1093/brain/aww035 26956421

[102] Weiss PH Ubben SD Kaesberg S Kalbe E Kessler J Liebig T. Where language meets meaningful action: a combined behavior and lesion analysis of aphasia and apraxia. Brain Struct Funct. (2016) 221:563–76. 10.1007/s00429-014-0925-3 25352157

[103] Manuel AL Radman N Mesot D Chouiter L Clarke S Annoni JM. Inter- and intrahemispheric dissociations in ideomotor apraxia: a large-scale lesion-symptom mapping study in subacute brain-damaged patients. Cereb Cortex (2013) 23:2781–9. 10.1093/cercor/bhs280 22989580

[104] Hermsdörfer J Li Y Randerath J Roby-Brami A Goldenberg G. Tool use kinematics across different modes of execution. Implications for action representation and apraxia. Cortex (2013) 49:184–99. 10.1016/j.cortex.2011.10.010 22176873

[105] Ohgami Y Matsuo K Uchida N Nakai T. An fMRI study of tool-use gestures: body part as object and pantomime. Neuroreport (2004) 15:1903–6. 10.1097/00001756-200408260-00014 15305134

[106] Rumiati RI Weiss PH Shallice T Ottoboni G Noth J Zilles K. Neural basis of pantomiming the use of visually presented objects. Neuroimage (2004) 21:1224–31. 10.1016/j.neuroimage.2003.11.017 15050550

[107] Johnson-Frey SH Newman-Norlund R Grafton ST. A distributed left hemisphere network active during planning of everyday tool use skills. Cereb Cortex (2005) 15:681–95. 10.1093/cercor/bhh169 15342430 PMC1364509

[108] Fridman EA Immisch I Hanakawa T Bohlhalter S Waldvogel D Kansaku K. The role of the dorsal stream for gesture production. Neuroimage (2006) 29:417–28. 10.1016/j.neuroimage.2005.07.026 16154363

[109] Imazu S Sugio T Tanaka S Inui T. Differences between actual and imagined usage of chopsticks: an fMRI study. Cortex (2007) 43:301–7. 10.1016/S0010-9452(08)70456-8 17533754

[110] Vingerhoets G Vandekerckhove E Honoré P Vandemaele P Achten E. Neural correlates of pantomiming familiar and unfamiliar tools: action semantics versus mechanical problem solving? Hum Brain Mapp. (2011) 32:905–18. 10.1002/hbm.21078 20629027 PMC6869918

[111] Vingerhoets G Acke F Alderweireldt AS Nys J Vandemaele P Achten E. Cerebral lateralization of praxis in right- and left-handedness: same pattern, different strength. Hum Brain Mapp. (2012) 33:763–77. 10.1002/hbm.21247 21500314 PMC6870330

[112] Vry MS Tritschler LC Hamzei F Rijntjes M Kaller CP Hoeren M. The ventral fiber pathway for pantomime of object use. Neuroimage (2015) 106:252–63. 10.1016/j.neuroimage.2014.11.002 25462791

[113] Goldenberg G Karnath HO. The neural basis of imitation is body part specific. J Neurosci. (2006) 26:6282–7. 10.1523/JNEUROSCI.0638-06.2006 16763035 PMC6675202

[114] Buxbaum LJ Shapiro AD Coslett HB. Critical brain regions for tool-related and imitative actions: a componential analysis. Brain (2014) 137(Pt 7):1971–85. 10.1093/brain/awu111 24776969 PMC4065019

[115] Dressing A Nitschke K Kümmerer D Bormann T Beume L Schmidt CS. Distinct contributions of dorsal and ventral streams to imitation of tool-use and communicative gestures. Cereb. Cortex (2018) 28:474–92. 10.1093/cercor/bhw383 27909000

[116] Binder E Dovern A Hesse MD Ebke M Karbe H Saliger J. Lesion evidence for a human mirror neuron system. Cortex (2017) 90:125–37. 10.1016/j.cortex.2017.02.008 28391066

[117] Martin M Beume L Kümmerer D Schmidt CS Bormann T Dressing A. Differential roles of ventral and dorsal streams for conceptual and production-related components of tool use in acute stroke patients. Cereb Cortex (2016) 26:3754–71. 10.1093/cercor/bhv179 26271112

[118] Schlaug G Knorr U Seitz R. Inter-subject variability of cerebral activations in acquiring a motor skill: a study with positron emission tomography. Exp Brain Res. (1994) 98:523–34. 10.1007/BF00233989 8056072

[119] Binkofski F Buccino G Posse S Seitz RJ Rizzolatti G Freund H. A fronto-parietal circuit for object manipulation in man: evidence from an fMRI-study. Eur J Neurosci. (1999) 11:3276–86. 10.1046/j.1460-9568.1999.00753.x 10510191

[120] Harrington DL Rao SM Haaland KY Bobholz JA Mayer AR Binderx JR. Specialized neural systems underlying representations of sequential movements. J Cogn Neurosci. (2000) 12:56–77. 10.1162/08989290051137602 10769306

[121] Haslinger B Erhard P Weilke F Ceballos-Baumann AO Bartenstein P Gräfin von Einsiedel H. The role of lateral premotor-cerebellar-parietal circuits in motor sequence control: a parametric fMRI study. Brain Res Cogn Brain Res. (2002) 13:159–68. 10.1016/S0926-6410(01)00104-5 11958958

[122] Decety J Perani D Jeannerod M Bettinardi V Tadary B Woods R. Mapping motor representations with positron emission tomography. Nature (1994) 371:600–2. 10.1038/371600a0 7935791

[123] Stephan KM Fink GR Passingham RE Silbersweig D Ceballos-Baumann AO Frith CD. Functional anatomy of the mental representation of upper extremity movements in healthy subjects. J Neurophysiol. (1995) 73:373–86. 10.1152/jn.1995.73.1.373 7714579

[124] Grafton ST Arbib MA Fadiga L Rizzolatti G. Localization of grasp representations in humans by positron emission tomography. 2. Observation compared with imagination. Exp Brain Res. (1996) 112:103–11. 10.1007/BF00227183 8951412

[125] Seitz RJ Roland PE Leaning of sequential finger movements in man: a combined kinematic and positron emission tomography (PET) study. Eur J Neurosci. (1992) 4:154–65. 10.1111/j.1460-9568.1992.tb00862.x 12106378

[126] Grezes J Tucker M Armony J Ellis R Passingham RE Objects automatically potentiate action: an fMRI study of implicit processing, Eur. J Neurosci. (2003) 17:2735–40. 10.1046/j.1460-9568.2003.02695.x 12823480

[127] Heuninckx S Wenderoth N Debaere F Peeters R Swinnen SP. Neural basis of aging: the penetration of cognition into action control. J Neurosci. (2005) 25:6787–96. 10.1523/JNEUROSCI.1263-05.2005 16033888 PMC6725362

[128] Hesse MD Thiel CM Stephan KE Fink GR. The left parietal cortex and motor intention: an event-related functional magnetic resonance imaging study, Neuroscience (2006) 140:1209–21. 10.1016/j.neuroscience.2006.03.030 16675134

[129] Naito E Ehrsson HH. Somatic sensation of hand-object interactive movement is associated with activity in the left inferior parietal cortex. J Neurosci. (2006) 26:3783–90. 10.1523/JNEUROSCI.4835-05.2006 16597731 PMC6674143

[130] Papadelis C Arfeller C Erla S Nollo G Cattaneo L Braun C. Inferior frontal gyrus links visual and motor cortices during a visuomotor precision grip force task. Brain Res. (2016) 1650:252–66. 10.1016/j.brainres.2016.09.011 27641995

[131] Varney NR Damasio H. Locus of lesion in impaired pantomime recognition. Cortex (1987) 23:699–703. 10.1016/S0010-9452(87)80061-8 3443005

[132] Kalénine S Buxbaum LJ Coslett HB. Critical brain regions for action recognition: lesion symptom mapping in left hemisphere stroke. Brain (2010) 133:3269–80. 10.1093/brain/awq210 20805101 PMC2965423

[133] Blakemore SJ Frith CD Wolpert DM. The cerebellum is involved in predicting the sensory consequences of action. NeuroReport (2001) 12:1879–84. 10.1097/00001756-200107030-00023 11435916

[134] Hashimoto Y Sakai KL. Brain activations during conscious self-monitoring of speech production with delayed auditory feedback: an fMRI study. Hum Brain Mapp. (2003) 20:22–8. 10.1002/hbm.10119 12953303 PMC6871912

[135] Leube DT Knoblich G Erb M Grodd W Bartels M Kircher TT. The neural correlates of perceiving one's own movements. Neuroimage (2003) 20:2084–90. 10.1016/j.neuroimage.2003.07.033 14683712

[136] Leube DT Knoblich G Erb M Kircher TTJ. Observing one's hand become anarchic: an fMRI study of action identification. Conscious Cogn. (2003) 12:597–608. 10.1016/S1053-8100(03)00079-5 14656503

[137] Leube DT Knoblich G Erb M Schlotterbeck P Kircher TTJ. The neural basis of disturbed efference copy mechanism in patients with schizophrenia. Cogn Neurosci. (2010) 1:111–7. 10.1080/17588921003646156 24168277

[138] Shimada S Hiraki K Oda I. The parietal role in the sense of self-ownership with temporal discrepancy between visual and proprioceptive feedbacks. Neuroimage (2005) 24:1225–32. 10.1016/j.neuroimage.2004.10.039 15670700

[139] David N Cohen MX Newen A Bewernick BH Shah NJ Fink GR. The extrastriate cortex distinguishes between the consequences of one's own and others' behavior. NeuroImage (2007) 36:1004–14. 10.1016/j.neuroimage.2007.03.030 17478105

[140] Farrer C Frey SH Van-Horn JD Tunik E Turk D Inati S. The angular gyrus computes action awareness representations. Cereb Cortex (2008) 18:254–61. 10.1093/cercor/bhm050 17490989

[141] Yomogida Y Sugiura M Sassa Y Wakusawa K Sekiguchi A Fukushima A. The neural basis of agency: an fMRI study. NeuroImage (2010) 50:198–207. 10.1016/j.neuroimage.2009.12.054 20026225

[142] Sperduti M Delaveau P Fossati P Nadel J. Different brain structures related to self- and external-agency attribution: a brief review and meta-analysis. Brain Struct Funct. (2011) 216:151–7. 10.1007/s00429-010-0298-1 21212978

[143] Kurayama T Matsuzawa D Komiya Z Nakazawa K Yoshida S Shimizu E. P50 suppression in human discrimination fear conditioning paradigm using danger and safety signals. Int J Psychophysiol. (2012) 84:26–32. 10.1016/j.ijpsycho.2012.01.004 22251449

[144] Backasch B Sommer J Klöhn-Saghatolislam F Müller MJ Kircher TT Leube DT. Dysconnectivity of the inferior frontal gyrus: implications for an impaired self-other distinction in patients with schizophrenia. Psychiatry Res. (2014) 223:202–9. 10.1016/j.pscychresns.2014.05.007 24976632

[145] Khalighinejad N Haggard P. Modulating human sense of agency with non-invasive brain stimulation. Cortex (2015) 69:93–103. 10.1016/j.cortex.2015.04.015 26004997

[146] van Kemenade BM Arikan BE Kircher T Straube B. The angular gyrus is a supramodal comparator area in action-outcome monitoring. Brain Struct Funct. (2017) 222:3691–703. 10.1007/s00429-017-1428-9 28439662

[147] Straube B Schülke R Drewing K Kircher T van Kemenade BM. Hemispheric differences in the processing of visual consequences of active vs. passive movements: a transcranial direct current stimulation study. Exp Brain Res. (2017) 235:3207–16. 10.1007/s00221-017-5053-x 28762054

[148] Craig AD. How do you feel–now? The anterior insula and human awareness. Nat Rev Neurosci. (2009) 10:59–70. 10.1038/nrn2555 19096369

[149] Buxbaum LJ Kyle KM Tang K Detre JA Neural substrates of knowledge of hand postures for object grasping and functional object use: evidence from fMRI. Brain Res. (2006) 30:175–85. 10.1016/j.brainres.2006.08.010 16962075

[150] Buxbaum LJ. Learning, remembering, and predicting how to use tools: distributed neurocognitive mechanisms: comment on osiurak and badets. Psychol Rev. (2017) 124:346–60. 10.1037/rev0000051 28358565 PMC5375056

[151] Jax SA Rosa-Leyra DL Buxbaum LJ. Conceptual- and production-related predictors of pantomimed tool use deficits in apraxia. Neuropsychologia (2014) 62:194–201. 10.1016/j.neuropsychologia.2014.07.014 25107676 PMC4167573

[152] Watson CE Buxbaum LJ. Uncovering the architecture of action semantics. J Exp Psychol Hum Percept Perform. (2014) 40:1832–48. 10.1037/a0037449 25045905 PMC4224273

[153] Goldenberg G Hagmann S. Tool use and mechanical problem solving in apraxia. Neuropsychologia (1998) 36:581–9. 10.1016/S0028-3932(97)00165-6 9723930

[154] Osiurak F Jarry C Allain P Aubin G Etcharry-Bouyx F Richard I. Unusual use of objects after unilateral brain damage: the technical reasoning model. Cortex (2009) 45:769–83. 10.1016/j.cortex.2008.06.013 19084221

[155] Osiurak F Jarry C Le Gall D. Grasping the affordances, understanding the reasoning: toward a dialectical theory of human tool use. Psychol Rev. (2010) 117:517–40. 10.1037/a0019004 20438236

[156] Osiurak F Jarry C Lesourd M Baumard J Le Gall D. Mechanical problem-solving strategies in left-brain damaged patients and apraxia of tool use. Neuropsychologia (2013) 51:1964–72. 10.1016/j.neuropsychologia.2013.06.017 23796703

[157] Goldenberg G Spatt J. The neural basis of tool use. Brain (2009) 132(Pt 6):1645–55. 10.1093/brain/awp080 19351777

[158] Jarry C Osiurak F Delafuys D Chauviré V Etcharry-Bouyx F Le Gall D. Apraxia of tool use: more evidence for the technical reasoning hypothesis. Cortex (2013) 49:2322–33. 10.1016/j.cortex.2013.02.011 23537602

[159] Osiurak F. What neuropsychology tells us about human tool use? The four constraints theory (4CT): mechanics, space, time, and effort. Neuropsychol Rev. (2014) 24:88–115. 10.1007/s11065-014-9260-y 24723242

[160] Reynaud E Lesourd M Navarro J Osiurak F. On the neurocognitive origins of human tool use: a critical review of neuroimaging data. Neurosci Biobehav Rev. (2016) 64:421–37. 10.1016/j.neubiorev.2016.03.009 26976352

[161] Baumard J Lesourd M Jarry C Merck C Etcharry-Bouyx F Chauviré V. Tool use disorders in neurodegenerative diseases: roles of semantic memory and technical reasoning. Cortex (2016) 82:119–32. 10.1016/j.cortex.2016.06.007 27376932

[162] Baumard J Lesourd M Remigereau C Jarry C Etcharry-Bouyx F Chauviré V Tool use in neurodegenerative diseases: planning or technical reasoning? J Neuropsychol. [Epub ahead of print]. (2017) 10.1111/jnp.12121 28455846

[163] Buchmann I Randerath J. Selection and application of familiar and novel tools in patients with left and right hemispheric stroke: psychometrics and normative data. Cortex (2017) 94:49–62. 10.1016/j.cortex.2017.06.001 28711817

[164] Lesourd M Osiurak F Navarro J Reynaud E. Involvement of the left supramarginal gyrus in manipulation judgment tasks: contributions to theories of tool use. J Int Neuropsychol Soc. (2017) 23:685–91. 10.1017/S1355617717000455 28625209

[165] Osiurak F Badets A. Use of tools and misuse of embodied cognition: reply to Buxbaum (2017). Psychol Rev. (2017) 124:361–8. 10.1037/rev0000065 28358566

[166] Goldenberg G Laimgruber K Hermsdörfer J. Imitation of gestures by disconnected hemispheres. Neuropsychologia (2001) 39:1432–43. 10.1016/S0028-3932(01)00062-8 11585611

[167] Goldenberg G. (2001). Imitation and matching of hand and finger postures. Neuroimage 14(1 Pt 2):S132–6. 10.1006/nimg.2001.0820 11373144

[168] Goldenberg G. Apraxia and the parietal lobes. Neuropsychologia (2009) 47:1449–59. 10.1016/j.neuropsychologia.2008.07.014 18692079

[169] Pelgrims B Andres M Olivier E. Double dissociation between motor and visual imagery in the posterior parietal cortex. Cereb Cortex (2009) 19:2298–307. 10.1093/cercor/bhn248 19168666

[170] Pelgrims B Michaux N Olivier E Andres M. Contribution of the primary motor cortex to motor imagery: a subthreshold TMS study. Hum Brain Mapp. (2011) 32:1471–82. 10.1002/hbm.21121 21077146 PMC6870368

[171] Andres M Pelgrims B Olivier E. Distinct contribution of the parietal and temporal cortex to hand configuration and contextual judgements about tools. Cortex (2013) 49:2097–105. 10.1016/j.cortex.2012.11.013 23313011

[172] Palluel-Germain R Jax SA Buxbaum LJ. Visuo-motor gain adaptation and generalization following left hemisphere stroke. Neurosci Lett. (2011) 498:222–6. 10.1016/j.neulet.2011.05.015 21605626 PMC3119783

[173] Rüther NN Tettamanti M Cappa SF Bellebaum C. Observed manipulation enhances left fronto-parietal activations in the processing of unfamiliar tools. PLoS ONE (2014) 9:e99401. 10.1371/journal.pone.0099401 24911053 PMC4049811

[174] Yang J. The influence of motor expertise on the brain activity of motor task performance: a meta-analysis of functional magnetic resonance imaging studies. Cogn Affect Behav Neurosci. (2015) 15:381–94. 10.3758/s13415-014-0329-0 25450866

[175] Hermsdorfer J Bienkiewicz M Cogollor JM Russel M Jean-Baptiste E Parekh M CogWatch-Automated Assistant and rehabilitation of stroke-induced action disorders in the home environment. In: Harris D editor. Applications and Services, 8020. Berlin: Springer-Verlag (2013), 343–50.

[176] Mihailidis A Boger JN Craig T Hoey J. The COACH prompting system to assist older adults with dementia through handwashing: an efficacy study. BMC Geriatr (2008) 8:28. 10.1186/1471-2318-8-28 18992135 PMC2588599

[177] Pastorino M Fioravanti A Arredondo MT Cogollor JM Rojo J Ferre M. Preliminary evaluation of a personal healthcare system prototype for cognitive eRehabilitation in a living assistance domain. Sensors (Basel). (2014) 14:10213–33. 10.3390/s140610213 24922452 PMC4118340

[178] Ambron E Miller A Kuchenbecker KJ Buxbaum LJ Coslett HB. Immersive low-cost virtual reality treatment for phantom limb pain: evidence from two cases. Front Neurol. (2018) 9:67. 10.3389/fneur.2018.00067 29515513 PMC5825921

[179] Villiger M Bohli D Kiper D Pyk P Spillmann J Meilick B. Virtual reality-augmented neurorehabilitation improves motor function and reduces neuropathic pain in patients with incomplete spinal cord injury. Neurorehabil Neural Repair. (2013) 27:675–83. 10.1177/1545968313490999 23757298

[180] Pazzaglia M Galli G. Translating novel findings of perceptual-motor codes into the neuro-rehabilitation of movement disorders. Front Behav Neurosci. (2015) 9:222. 10.3389/fnbeh.2015.00222 26347631 PMC4543860

[181] Ertelt D Small S Solodkin A Dettmers C McNamara A Binkofski F. Action observation has a positive impact on rehabilitation of motor deficits after stroke. Neuroimage (2007) 36(Suppl. 2):T164–73. 10.1016/j.neuroimage.2007.03.043 17499164

[182] Page SJ Levine P Sisto S Johnston MV. A randomized efficacy and feasibility study of imagery in acute stroke. Clin Rehabil. (2001) 15:233–40. 10.1191/026921501672063235 11386392

[183] Sayegh PF Gorbet DJ Hawkins KM Hoffman KL Sergio LE. The contribution of different cortical regions to the control of spatially decoupled eye-hand coordination. J Cogn Neurosci. (2017) 29:1194–211. 10.1162/jocna01111 28253075

[184] Brovelli A Chicharro D Badier JM Wang H Jirsa V. Characterization of cortical networks and corticocortical functional connectivity mediating arbitrary visuomotor mapping. J Neurosci. (2015) 35:12643–58. 10.1523/JNEUROSCI.4892-14.2015 26377456 PMC6795203

[185] Renzi C Ricciardi E Bonino D Handjaras G Vecchi T Pietrini P. The effects of visual control and distance in modulating peripersonal spatial representation. PLoS ONE (2013) 8:e59460. 10.1371/journal.pone.0059460 23555037 PMC3598753

[186] Jax SA Buxbaum LJ Lie E Coslett HB. More than (where the target) meets the eyes: disrupted visuomotor transformations in optic ataxia. Neuropsychologia (2009) 47:230–8. 10.1016/j.neuropsychologia.2008.07.023 18725238 PMC2678860

[187] Striemer C Locklin J Blangero A Rossetti Y Pisella L Danckert J. Attention for action? Examining the link between attention and visuomotor control deficits in a patient with optic ataxia. Neuropsychologia (2009) 47:1491–9. 10.1016/j.neuropsychologia.2008.12.021 19154751

[188] Cavina-Pratesi C Ietswaart M Humphreys GW Lestou V Milner AD. Impaired grasping in a patient with optic ataxia: primary visuomotor deficit or secondary consequence of misreaching? Neuropsychologia (2010) 48:226–34. 10.1016/j.neuropsychologia.2009.09.008 19766131

